# Dysregulated mitochondrial energy metabolism drives the progression of mucosal field effects to invasive bladder cancer

**DOI:** 10.1002/path.6474

**Published:** 2025-09-25

**Authors:** Sangkyou Lee, Sung Yun Jung, Pawel Kuś, Jolanta Bondaruk, June Goo Lee, Roman Jaksik, Nagireddy Putluri, Khanh N Dinh, David Cogdell, Huiqin Chen, Yishan Wang, Jiansong Chen, Neema Navai, Colin Dinney, Cathy Mendelsohn, David McConkey, Richard R Behringer, Charles C Guo, Peng Wei, Marek Kimmel, Bogdan Czerniak

**Affiliations:** ^1^ Department of Pathology The University of Texas MD Anderson Cancer Center Houston TX USA; ^2^ Department of Molecular and Cellular Biology Baylor College of Medicine Houston TX USA; ^3^ Department of Systems Biology and Engineering Silesian University of Technology Gliwice Poland; ^4^ Metabolomics Core Baylor College of Medicine Houston TX USA; ^5^ Irving Institute for Cancer Dynamics and Department of Statistics Columbia University New York NY USA; ^6^ Department of Biostatistics The University of Texas MD Anderson Cancer Center Houston TX USA; ^7^ Department of Urology The University of Texas MD Anderson Cancer Center Houston TX USA; ^8^ Department of Urology, Genetics & Development and Pathology Columbia University New York NY USA; ^9^ Johns Hopkins Greenberg Bladder Cancer Institute Johns Hopkins University Baltimore MD USA; ^10^ Department of Genetics The University of Texas MD Anderson Cancer Center Houston TX USA; ^11^ Department of Statistics Rice University Houston TX USA

**Keywords:** bladder cancer, mutational landscape, time modeling, dysregulation of mitochondrial energy metabolism

## Abstract

Multiplatform mutational and gene expression profiling complemented with proteomic and metabolomic spatial mapping were used on the whole‐organ scale to identify the molecular profile of bladder cancer evolution from field effects. Analysis of the mutational landscape identified three types of mutations, referred to as α, β, and γ. Time modeling of the mutations revealed that carcinogenesis may span 30 years and can be divided into dormant and progressive phases. The α mutations developed in the dormant phase. The progressive phase lasted 5 years and was signified by expanding β mutations, but it was driven to invasive cancer by γ mutations. The mutational landscape emerged on a background of disorganized urothelial differentiation, activated epithelial‐mesenchymal transition, and enhanced immune infiltration with T‐cell exhaustion. Complex dysregulation of mitochondrial energy metabolism with downregulation of oxidative phosphorylation emerged as the leading mechanism driving the progression of mucosal field effects to invasive cancer. © 2025 The Author(s). *The Journal of Pathology* published by John Wiley & Sons Ltd on behalf of The Pathological Society of Great Britain and Ireland.

## Introduction

The molecular mechanisms that initiate carcinogenesis involve microscopically normal‐appearing tissue and are collectively referred to as field effects [1, 2]. Their characterization may facilitate the development of early detection, prevention, and treatment strategies for intercepting carcinogenesis in its early phases before it progresses to clinically aggressive and often uncurable disease. Understanding of these initiating events is not possible unless they are analyzed in the geographic spatial frame of mucosal changes in an entire organ. Bladder cancer is a particularly useful model for such studies, as it develops in the epithelial lining (urothelium) of an anatomically simple organ, facilitating combined geographic microscopic and multiplatform genomic mapping. We and others recently characterized this field cancerization using genomics [3, 4, 5, 6, 7]. Those studies revealed that regionally restricted clonal expansion occurs in areas of the urothelium that appear phenotypically normal and that these changes are associated with mutations of cancer driver genes, particularly those that regulate the chromatin structure [3, 4, 8]. These changes are associated with alterations in RNA expression that appear to disrupt innate immunity among other pathways and cause T‐cell exhaustion [3, 8]. However, very little is known about how these genomic changes alter urothelial biology, and acquiring this knowledge requires a deeper understanding of the downstream effects on protein expression and metabolism. Herein we provide the first description of proteomic and metabolomic profiles of bladder cancer evolution from mucosal field effects in the context of their mutational landscape and gene expression profile on the whole‐organ scale.

## Materials and methods

### Ethics approval and patient selection

Human samples and clinical data were collected according to laboratory protocols approved by the Institutional Review Board of The University of Texas MD Anderson Cancer Center. Whole‐organ histologic, genomic, proteomic, and metabolomic mapping was performed using a radical cystectomy sample obtained from a 92‐year‐old white male patient with high‐grade urothelial carcinoma (UC) that invaded into the muscularis propria (T3), with no evidence of metastatic disease in the pelvic lymph nodes, as described previously [3, 4]. The patient was a retired navy serviceman and businessman with 42 pack‐years of cigarette smoking. Validation analyses were performed on The Cancer Genome Atlas (TCGA) cohort (*n* = 408) [9]. The TCGA mutational data were downloaded in the mutation annotation format (MAF) from the GDAC website (http://gdac.broadinstitute.org). The level 3 RNASeq data were downloaded using the R package TCGAGeneReport (v2.3, https://github.com/MD-Anderson-Bioinformatics/GeneSurvey).

### Whole‐exome sequencing and data analysis

Whole‐exome sequencing was performed with 37 geographically annotated mucosal DNA samples and normal genomic DNA as a reference. DNA sequencing and downstream data analysis were performed as outlined in recent cancer sequencing project reports. In brief, whole‐exome sequencing was performed using an Illumina NovaSeq 6,000 sequencer (Illumina, San Diego, CA, USA) with a high‐output flow cell and a mean (±SD) coverage across the samples of 300× ± 85.

### Mutational analysis

Analysis of mutational signatures was performed as previously described [3]. In brief, nonsilent mutations, which were present in at least one mucosal sample with the substitutions C > A, C > G, C > T, T > A, T > C, and T > G, were used to analyze their distribution in three groups of samples corresponding to normal urothelium (NU)/low‐grade intraepithelial neoplasia (LGIN), low‐grade intraepithelial neoplasia (HGIN), and UC as described previously [10, 11, 12]. Fisher's exact test was used to test the null hypothesis that they are equally distributed in the three groups of samples. The genomic context of single nucleotide variants (SNVs), including the two flanking bases on the 5’ and 3’ sides of each SNV, was assembled and included 96 mutational patterns. The frequency of any fingerprints between groups of mucosal samples was determined using the Wilcoxon rank sum test. The Benjamini–Hochberg method was used to assess the false discovery rate (FDR) [13].

### Phylogenetic analysis and modeling of bladder cancer evolution

A phylogenetic tree was constructed by calculating the Hamming distances among the mucosal samples using a matrix of all nonsilent and silent mutations present in at least one mucosal sample by applying the maximum parsimony algorithm, as previously described [3]. To reconstruct the time of evolution from mucosal field effects to bladder cancer, a parsimonious time‐continuous Markov branching process with immigration was employed, as described previously [14].

### RNA‐seq and data analysis

The RNA integrity was assessed using a 2,100 Bioanalyzer (Agilent, Santa Clara, CA, USA). RNA concentration was determined using Quant‐iT RiboGreen RNA Assay Kit (Thermo Fisher Scientific, Waltham, MA, USA) following the manufacturer's protocol. RNA samples meeting a quantity threshold of 1 μg and with an RNA integrity number of at least three were analyzed in the Advanced Technology Genomics Core at MD Anderson. Prior to RNA library construction, ribosomal RNA was removed from total RNA preparations, and cDNA synthesis using oligo(dT) and random hexamers was performed. The library was made up of random fragments representing the entire sample. It was created by shearing DNA into 150–400 base fragments that were ligated to specific adapters. Following a sample cleanup step, the resulting library was quantified using quantitative polymerase chain reaction and checked for quality using a TapeStation (Agilent). The analyses were performed with 37 RNA geographically annotated mucosal samples and three sex‐matched control NU suspensions, which were prepared from the ureters in nephrectomy samples that were free of urothelial neoplasia [15].

### Mass spectrometry‐based proteome profiling

Proteome profiling was performed using 35 geographically annotated mucosal samples and three sex‐matched control NU suspensions prepared from the ureters in nephrectomy samples that were free of urothelial neoplasia. Global proteome profiling was processed as previously described, with some modifications [16, 17].

### Targeted metabolomic analysis

Targeted metabolomic analysis was performed using 42 geographically annotated mucosal samples and three sex‐matched control NU suspensions prepared from the ureters in nephrectomy samples that were free of urothelial neoplasia. Human bladder mucosal and tumor tissue lysates were used for metabolite extraction. Lysates from the urothelium harvested from normal human ureters in nephrectomy samples of patients with renal cancer, but without evidence of urothelial neoplasia, were used as a reference. A mouse liver pool was used for quality control and was combined with 750 μl of internal standard mix. For extraction of metabolites, geographically mapped cell suspensions of the cystectomy and control reference samples were defrosted and processed using the liquid–liquid extraction method [18, 19, 20]. The extracted total metabolite samples were analyzed through high‐throughput liquid chromatography‐MS techniques previously described [18, 19].

### Integrative analysis of pathways

To identify molecular pathways involved in the development of bladder cancer, Ingenuity Pathway Analysis was performed, focusing on pathways dysregulated in the field effects and whose dysregulation continued in progression to HGIN and UC, referred to as monotonic dysregulation [21, 22]. Abnormally expressed and dysmethylated genes as well as mutated genes from clusters α and β were used in these analyses.

## Results

### Preparation of a whole‐organ cystectomy for spatial mapping

**Figure 1:**
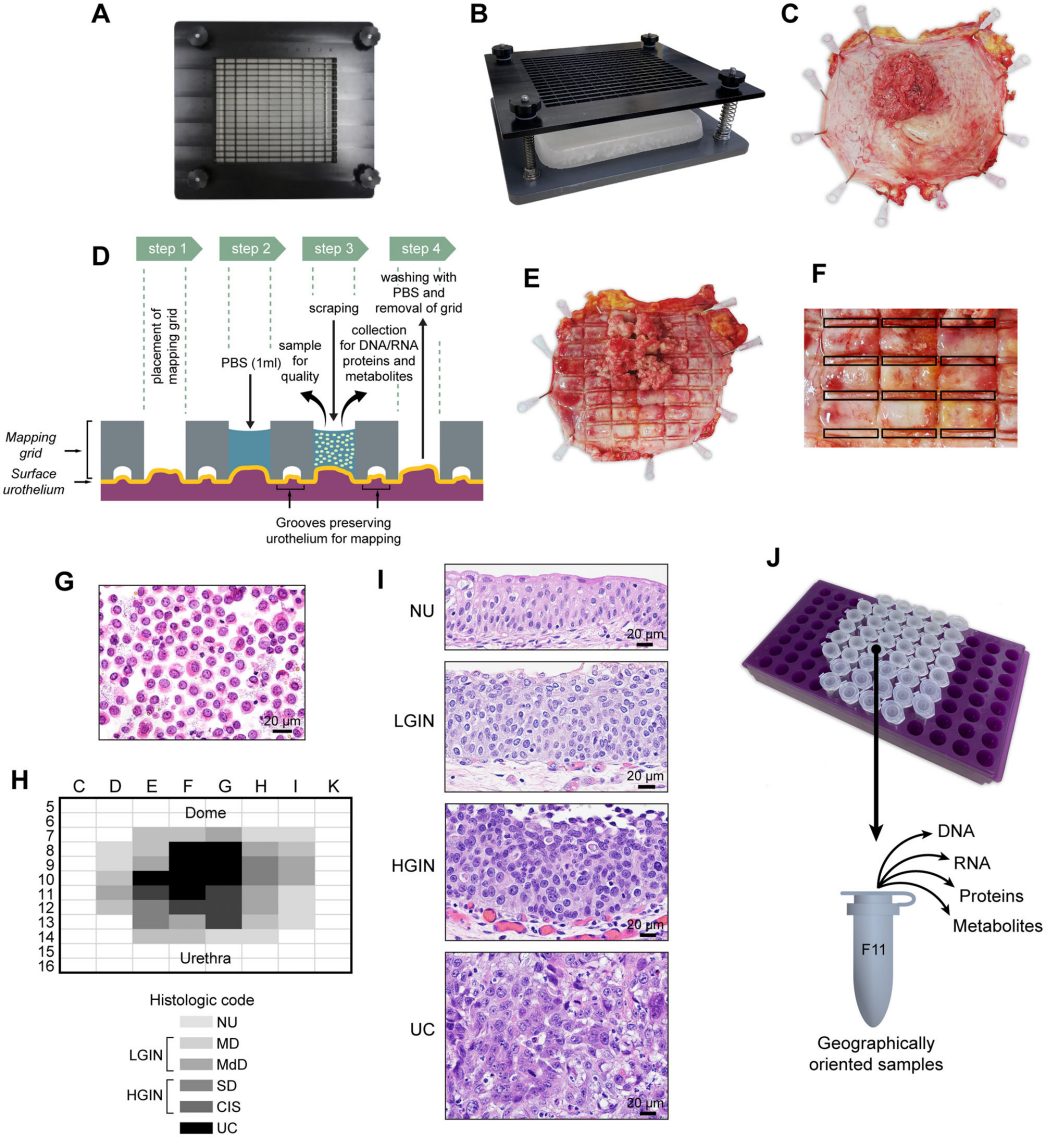
Preparation of whole‐organ maps for multiplatform genomic, proteomic, and metabolomic profiling. (A) Top view of the mapping grid for whole‐organ sampling. (B) Oblique view of the mapping grid. (C) Photograph of an open cystectomy sample pinned to a paraffin block showing a fungating tumor involving the posterior bladder wall. (D) Diagram of the details of the mapping grid preserving the urothelium for histologic mapping and permitting simultaneous DNA/RNA and protein extraction. PBS, phosphate‐buffered saline. (E) The open cystectomy sample shown in (C) with impressions of the mapping grid for histologic sampling. (F) Enlarged view of the mucosal area of the open cystectomy sample with the impression of the mapping grid showing the sampling pattern for histologic mapping of the mucosa. (G) A urothelial single‐cell suspension after sample collection from geographically mapped mucosal areas used for DNA/RNA and protein extraction. (H) A whole‐organ histologic map prepared by sampling of the entire bladder mucosa in the cystectomy sample shown in (C). MD, mild dysplasia; MdD, moderate dysplasia; SD, severe dysplasia. (I) Representative microscopic images corresponding to normal urethelium (NU), low‐grade intraepithelial neoplasia (LGIN), high‐grade intraepithelial neoplasia (HGIN), and urothelial carcinoma (UC). (J) Geographically mapped urothelial cell suspensions corresponding to the histologic map shown in H used for DNA/RNA and protein extraction.

To molecularly characterize the evolution of bladder cancer from mucosal field effects on the whole‐organ scale, we collected geographically annotated mucosal samples from a representative cystectomy sample from a patient with invasive bladder cancer (Figure 1A−J). For whole‐organ mapping, we opened the resected human bladder along the anterior wall and pinned it down to a paraffin block. We then applied a mapping grid, which separated the mucosal areas into 1 × 2‐cm wells, allowing DNA and protein to be extracted while simultaneously preserving the urothelium for microscopic inspection, which we used in reconstructing a histologic map of the entire bladder mucosa and invasive cancer. We microscopically classified the samples as NU; *in situ* preneoplastic conditions, referred to as LGIN and HGIN, respectively; and invasive UC. Additionally, we examined geographically oriented samples using bulk whole‐exome DNA and RNA sequencing, proteomic sequencing, and targeted metabolomic analysis. We used germline DNA from peripheral blood samples as a reference for DNA sequencing. Finally, we used microscopically NU harvested from ureters in nephrectomy samples obtained from patients with renal cancer but without urothelial neoplasia as a reference for RNA sequencing, proteomic, and metabolomic analyses.

### Mutational landscape of field effects and their evolution to carcinoma

**Figure 2:**
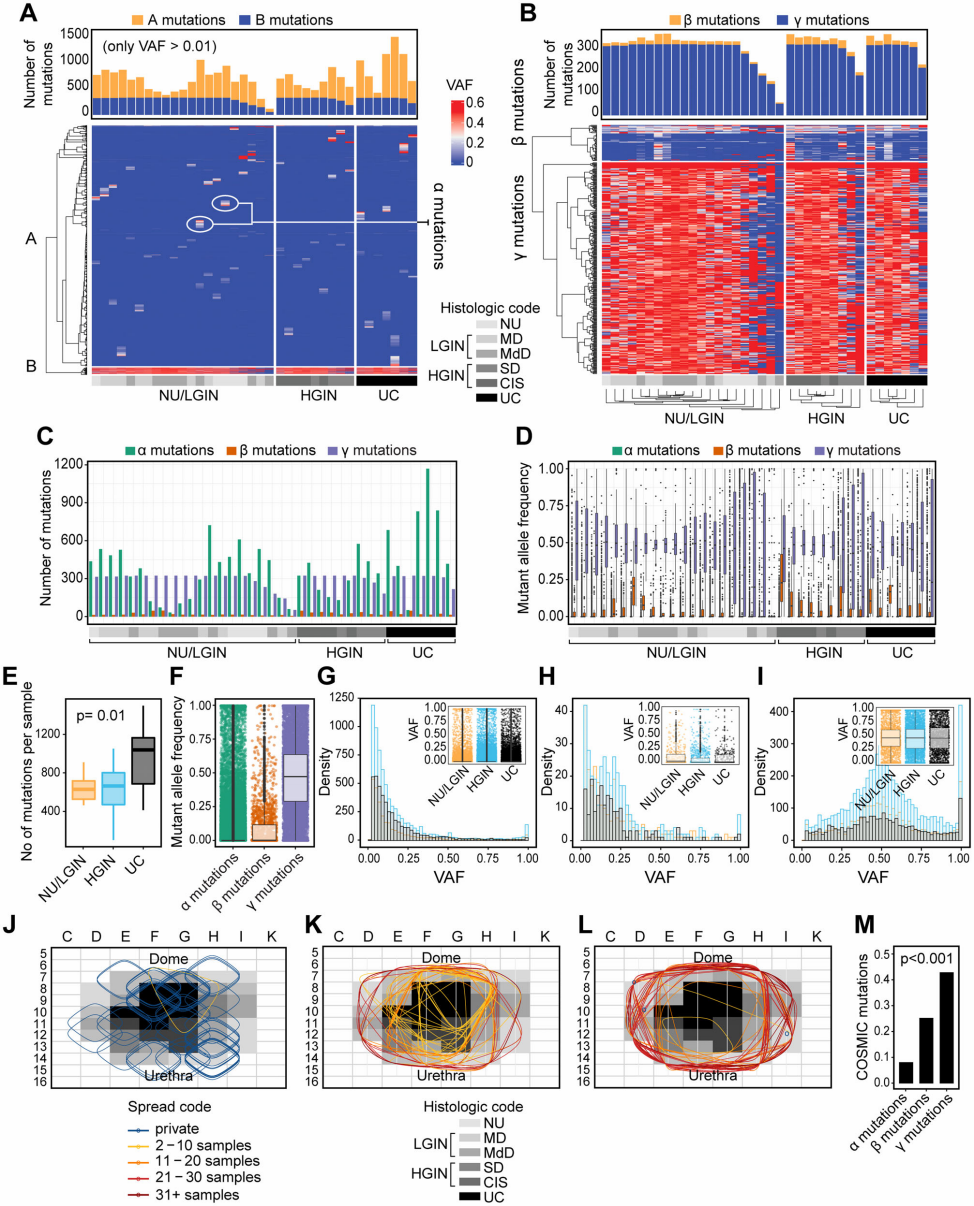
Mutational landscape of bladder cancer evolution from field effects. (A) Heatmap of nonsilent mutations showing variant allele frequencies (VAFs) in individual mucosal samples. The numbers of mutations in individual mucosal samples are shown in the top diagram. (B) Heatmap of VAFs ≥ 0.01 in genes showing variant alleles in at least three mucosal samples. The numbers of β and γ mutations in individual mucosal samples are shown in the top diagram. An enlarged view of panel B is available in the supplementary material, Figure S15. (C) The numbers of α, β, and γ mutations in individual mucosal samples. (D) VAFs of α, β, and γ mutations in individual mucosal samples. An enlarged view of panel D is available in the supplementary material, Figure S15. (E) Boxplot of the number of mutations in mucosal samples classified as normal urethelium (NU), low‐grade intraepithelial neoplasia (LGIN), high‐grade intraepithelial neoplasia (HGIN), and urothelial carcinoma (UC). (F) VAFs of α, β, and γ mutations. (G) Histogram showing the clonality of VAFs of α mutations. Inset, boxplot of VAFs of α mutations in three groups of samples corresponding to NU/LGIN, HGIN, and UC. (H) Histogram showing the clonality of VAFs of β mutations. Inset, boxplot of VAFs of β mutations in three groups of samples corresponding to NU/LGIN, HGIN, and UC. (I) Histogram showing the clonality of VAFs of γ mutations. Inset, boxplot of VAFs of γ mutations in three groups of samples corresponding to NU/LGIN, HGIN, and UC. (J) Spatial distribution of 50 randomly selected α mutations superimposed on a histologic map of a cystectomy. (K) Spatial distribution of β mutations superimposed on a histologic map of a cystectomy. (L) Spatial distribution of 50 randomly selected γ mutations superimposed on a histologic map of a cystectomy. (M) Proportions of COSMIC driver mutations in α, β, and γ mutations. In G, H, and L, NU samples are shown in orange, HGIN samples are shown in blue, and UC samples are shown in black. Also, in J, K, and L, the color‐coded ovals represent mutation spread.

Whole‐exome sequencing of DNA from geographically mapped mucosal samples identified nonsynonymous variant alleles in 12,764 genomic loci (12,022 SNVs, 450 inserts, and 292 deletions) (supplementary material, Table S1). A heatmap of the geographic distribution and variant allele frequencies (VAFs) of these mutations in individual mucosal samples is shown in Figure 2A. Based on their VAFs and geographic distribution, we separated these mutations into two major groups. The first group (cluster A) comprised mutations with low VAFs confined to individual mucosal samples, referred to as α mutations (supplementary material, Table S2). The second group (cluster B) comprised mutations expanding across the bladder mucosa. We further divided cluster B mutations into two subgroups, referred to as β and γ (supplementary material, Tables S3, S4; Figure 2B). Mutations in cluster β expanded across the bladder mucosa, but their VAFs were consistently low (<20%) and did not increase with progression to HGIN and UC. On the other hand, mutations in cluster γ formed a plaque involving large areas of bladder mucosa with consistently high VAFs (>20%). Overall, based on the geographic distribution and VAFs, we identified three distinct types of mutations, referred to as α, β, and γ (supplementary material, Table S1 and Figure 2A–D). The α mutations were the most frequent (12,431), comprising 11,698 SNVs, 448 insertions, and 285 deletions. We observed only 54 β mutations, with 51 SNVs, one insertion, and two deletions. Finally, we observed 324 γ mutations, with 315 SNVs, two insertions, and seven deletions. Although the VAFs of the γ mutations were high in all three groups of samples corresponding to NU/LGIN, HGIN, and UC, we saw a major increase in the number of mutations with progression to HGIN and UC (Figure 2C–F). Most of those mutations were of the γ type.

To address the issue of preferential selection of mutations and their potential driver role, we analyzed their spatial distribution and VAFs in different fields of the map described above (supplementary material, Figure S1A–D). We focused on the distribution patterns for α, β, and γ mutations to test the hypothesis that some of these mutations may be under positive selection, whereas others are mere hitchhikers (Figure 2G−I). First, we classified the mutations as private (present in only one field), regional (present in 2–10 or 11–20 fields), and widespread (present in 21–30 or >30 fields) mutations. The α mutations were almost exclusively private, whereas the γ mutations were almost exclusively widespread. Some of the β mutations were regional, whereas others were widespread. In addition to being private, the α mutations had right‐skewed VAF distributions typical of proliferating cells with neutral mutations (Figure 2G,J; supplementary material, Figure S1A) [23]. Similarly, the VAFs of β mutations were right‐skewed, but their spread was at least regional, involving several mucosal samples (Figure 2H,K; supplementary material, Figure S1B). The VAFs of the widespread γ mutations were binomially distributed in most fields, signifying a secondary clone that acquired a positive selective advantage (Figure 2I,L; supplementary material, Figure S1C) [23]. In some fields, the VAF distributions for γ mutations were almost uniform, which is likely a sign of a widespread genome copy number dysregulation (supplementary material, Figure S1C). The VAFs of β mutations in individual samples were highly irregular, in general; the VAFs had more right‐skewed distribution patterns in some fields, but binomial distributions in others. This pattern suggests a mixture of clones with different proliferative advantages. We filtered α, β, and γ mutations for their COSMIC (Catalogue Of Somatic Mutations In Cancer mutations) [24] (supplementary material, Tables S5–S7 and supplementary material, Figure S2A,B). We then analyzed their affected pathways (supplementary material, Figure S3A–C). For α mutations that were restricted to small mucosal areas, we performed these analyses for individual mucosal samples (supplementary material, Figure S3D). Overall, we observed a progressive increase of COSMIC driver mutations among α, β, and γ mutations (Figure 2M). The α mutations showed the expected diversity of their affected pathways among the individual mucosal samples (supplementary material, Figure S3D). However, some of the pathways appeared to be repeatedly involved with phagosome formation, cardiomyopathy, and calcium signaling, as well as sildenafil and CREB signaling being involved in over 70% of individual mucosal samples affected by α mutations (supplementary material, Figure S3E). Dysregulation of RHO GTPase signaling was evident in over 30% of mucosal areas with α mutations. Interestingly, multiple pathways involved in oncogenic transformation, including those attributed in genomic instability such as BRCA1, as well as multiple aspects of p53 dysregulation, were synchronous with the advent of β mutations (supplementary material, Figure S3B). These changes were complemented by dysregulation of multiple pathways involved in cell biology, including reelin signaling, lipoprotein metabolism, nerve growth factor processing, and various aspects of cell differentiation related to γ mutations (supplementary material, Figure S3C).

### Mechanisms of mutagenesis involved in bladder cancer evolution from field effects

To characterize the mutagenesis signatures in the evolution of bladder cancer from field effects, we first analyzed six single‐based nucleotide substitutions (C > A, C > G, C > T, T > A, T > C, and T > G) and their context motifs in all mucosal samples (supplementary material, Figure S4A–C). The frequency of C > T substitutions increased at the transition from NU/LGIN to HGIN and UC, with significant changes in 12 mutational signatures. Signatures 1, 6, 12, 20, and 24 appeared to be the most dominant, with signature 1 having the highest weight scores (supplementary material, Figure S4D–F). To evaluate the contributions of individual mutagenesis patterns to the mucosal mutational landscape, we performed bootstrapping and calculated the *p* value to assess their significance (*p* < 0.005 was considered significant). This approach confirmed the dominance of signatures 1 and 6 (supplementary material, Figure S4G). Analysis of α, β, and γ mutations identified a significant increase in C > T substitutions in γ mutations (supplementary material, Figure S4H). Similarly, these three groups of mutations were associated with distinct mutational signatures, confirming their development through distinct mutational mechanisms (supplementary material, Figure S4I–K).

Since our map showed the preferential involvement of the mutational signatures 1 and 6 attributed to APOBEC deficiency and genomic instability, respectively, we validated their involvement in the TCGA cohort (*n* = 408) (supplementary material, Figure S4L–N) [25, 26]. This showed that our case corresponds to a subset of bladder tumors with coinvolvement of the mutational signatures 1 and 6. Because the analysis of mutational signatures showed the involvement of signature 6 in our cystectomy specimen, we analyzed the case for the potential germline mutations of cancer predisposing genes, including those involved in genomic instability and found no germline mutations.

### Modeling of bladder cancer evolution from field effects

**Figure 3:**
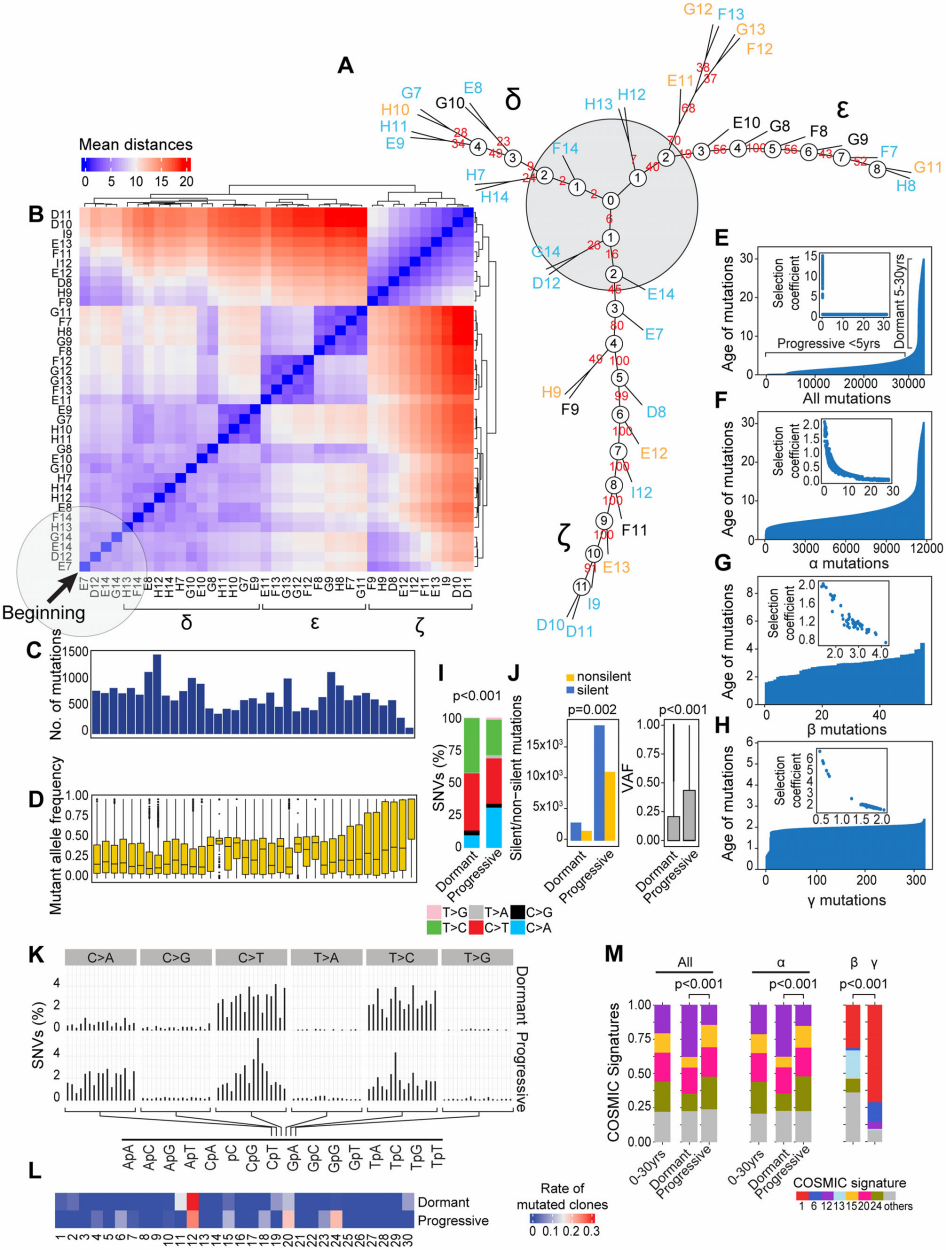
Modeling of bladder cancer evolution from its mutational landscape. (A) Parsimony analysis showing an evolutionary tree of expansion of successive clones of cells in the field effects corresponding to normal urethelium (NU), low‐grade intraepithelial neoplasia (LGIN) along three branches designated as δ, ε, and ζ. The hypothetical beginning of the process is designated as node 0 and depicted as in the gray circle. (B) Heatmap of genetic distances for successive clones with three clusters corresponding to branches δ, ε, and ζ in A. The beginning of carcinogenesis is shown in the lower left corner, indicated by the black arrow. (C) The numbers of mutations in individual cystectomy samples organized in the same order as in B. (D) VAFs in individual cystectomy samples organized in the same order as in B. Of note is the increase in VAFs in samples corresponding to branch ζ. (E) Ages of all synonymous and nonsynonymous mutations predicted by mathematical modeling. Inset: the selection coefficient in relation to the predicted mutation age. (F) Ages of α mutations predicted by mathematical modeling. Inset: the selection coefficients for α mutations. (G) Ages of β mutations predicted by mathematical modeling. Inset: the selection coefficients for β mutations. (H) Ages of γ mutations predicted by mathematical modeling. Inset: the selection coefficients for γ mutations. (I) Bar graph of nucleotide substitutions in the dormant and progressive phases of bladder carcinogenesis. (J) Left: numbers of silent and nonsilent mutations in the dormant and progressive phases, respectively, of bladder carcinogenesis. Right: VAFs in the dormant and progressive phases of bladder carcinogenesis. (K) Proportions of SNVs in nucleotide motifs for each category of substitution in the dormant and progressive phases of bladder carcinogenesis. (L) Weight scores for mutagenesis patterns in the dormant and progressive phases of bladder carcinogenesis. (M) Composition of the mutational signatures in α, β, and γ mutations in the dormant and progressive phases of bladder carcinogenesis.

To analyze the mutational pattern of clonal evolution of bladder cancer from field effects, we used all nonsilent and silent mutations to construct an evolutionary tree. This revealed a complex branching pattern corresponding to multiple waves of clonal expansion, with 4–11 nodes evolving along three distinct branches referred to as δ, ε, and ζ (Figure 3A). The process evolved from hypothetical node 0 in the center of the tree and neighboring nodes 1 and 2 of the three branches representing incipient events of carcinogenesis. The heatmap of the genetic distances among individual samples in Figure 3B shows a gradual evolution of the mutational landscape from the initiating events in the left lower corner, with three clusters of the mutational landscape corresponding to the three branches of the evolutionary tree. Branch ζ appeared to be the most mutationally active, exhibiting increased VAFs (Figure 3C,D).

To answer the question of how long bladder cancer takes to develop, we applied a mathematical modeling algorithm to a whole‐organ mutational landscape. We used successive waves of clonal evolution, applying a time‐continuous Markov branching process with immigrations [14]. This provided a time scale for cancer evolution from field effects based on parsimonious principles. Initially, we performed the analysis using all synonymous and nonsynonymous mutations (Figure 3E). These analyses demonstrated that cancer evolved from field effects over about 30 years and that the age‐related curve of mutations had a left‐skewed pattern, with the mutations gradually developing over nearly three decades. Using the time scale for mutations, we divided the process of tumor evolution into two major phases, referred to as dormant and progressive. The first, older dormant phase, in which mutations developed over about two decades, involved mutations that were characterized by low selection coefficients consistent with their marginal proliferative advantage. The second, more recent progressive phase was less than 5 years old and was characterized by a large number of mutations, with high selection coefficients consistent with their clonal expansion and high proliferative advantage. We then repeated the modeling analysis selectively using the α, β, and γ mutations (Figure 3F−H). These analyses demonstrated that the oldest of the α mutations started developing the earliest, which continued occurring over the 30 years of tumor history (Figure 3F). A large proportion of the mutations had low selection coefficients, consistent with a slight proliferative advantage and the fact that they involved small mucosal areas. The β type mutations emerged at the transition from dormant to progressive phase and occurred over a period shorter than 5 years (Figure 3G). A large proportion of these mutations exhibited increased selection coefficients consistent with a proliferative advantage and clonal expansion. The mutations of the γ type were the most recent, occurring over the last 2 years before cancer diagnosis (Figure 3H). These mutations were characterized by high selection coefficients consistent with a proliferative advantage and involvement over the last 2 years of the progressive phase. Overall, these results demonstrated that the dormant and progressive phases of bladder carcinogenesis were characterized by distinct involvement of α, β, and γ mutations.

To address the issue of potential distinctive involvement of different mutagenesis mechanisms in different phases of bladder carcinogenesis, we performed a detailed analysis of the mutational signatures in the dormant and progressive phases of the disease. These analyses demonstrated that progressive disease was characterized by an increased number of C > A substitutions combined with major increases in both silent and nonsilent mutation numbers and their VAFs (Figure 3I,J). Both phases of bladder carcinogenesis exhibited the involvement of distinct mutagenesis signatures, confirming the concept that distinct mutational mechanisms are switched on a background of dormant field effects driving the progression to clinically aggressive bladder cancer (Figure 3K,L). Similarly, α, β, and γ mutations exhibited different compositions of mutagenesis signatures, with α mutations having polyclonal heterogeneous involvement of different signatures. The emergence of β mutations at the transition to the progressive phase was associated with prominence of signature 1, which was followed by its dramatic expansion in γ mutations (Figure 3M).

### The RNA expression profile of bladder cancer evolution from field effects

**Figure 4:**
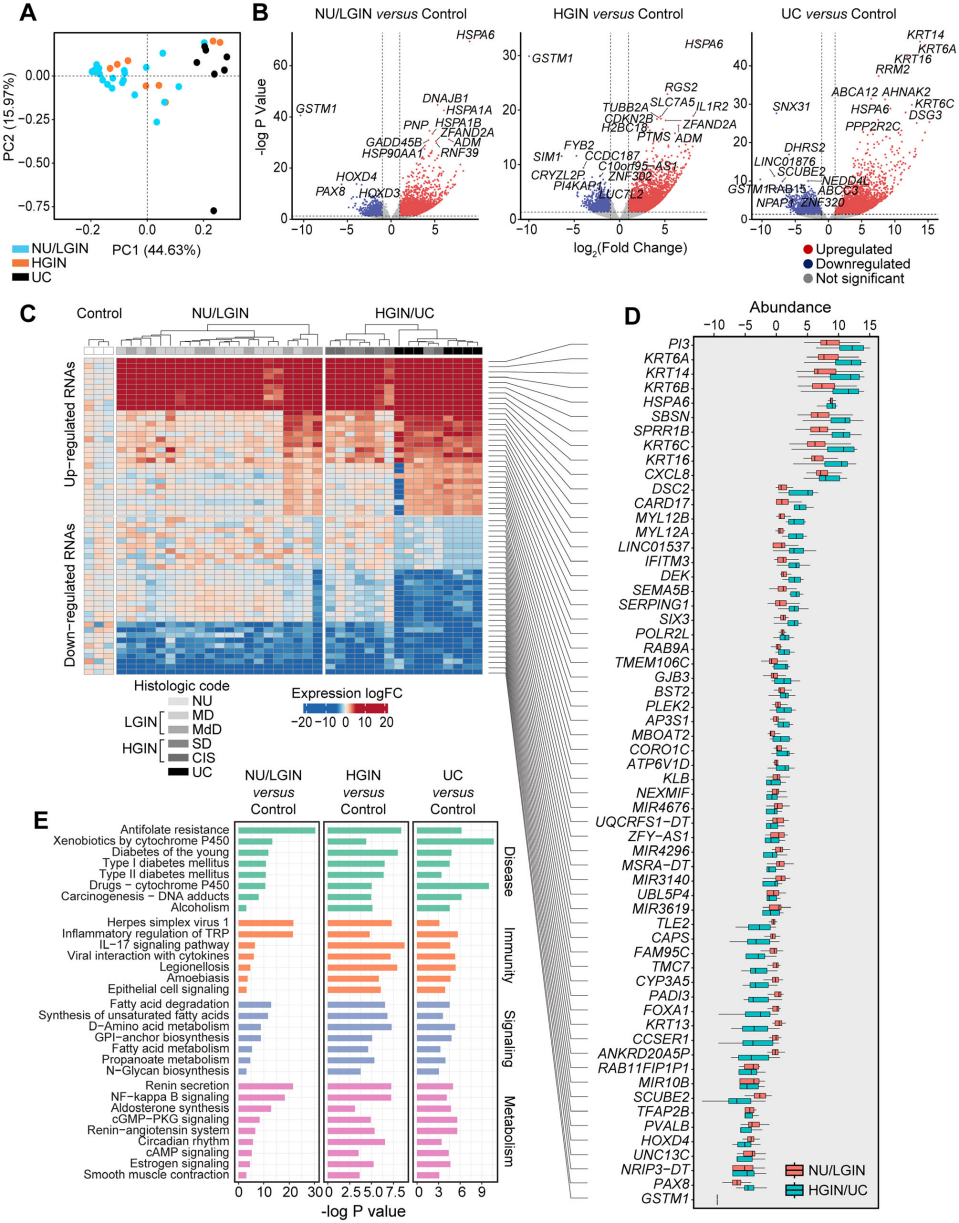
RNA‐seq–based gene expression profile of bladder cancer evolution from mucosal field effects. (A) PCA of gene expression data for all mucosal samples. (B) Volcano plots of RNA expression levels comparing log2 fold changes with −log *p* values in normal urethelium (NU)/low‐grade intraepithelial neoplasia (LGIN), high‐grade intraepithelial neoplasia (HGIN), and urothelial carcinoma (UC) samples versus those in control samples. (C) Heatmap of the top 30 upregulated and top 30 downregulated genes in mucosal samples. (D) Boxplot of the top 30 upregulated and top 30 downregulated genes in C depicting changes in NU/LGIN and HGIN/UC samples compared with control samples. (E) Monotonically dysregulated KEGG pathways showing −log *p* values for the comparison of NU/LGIN, HGIN, and UC samples with control samples.

We used whole‐transcriptome expression changes based on the results of RNA sequencing (RNA‐seq) to analyze the gene expression profile changes associated with the evolution of bladder cancer from mucosal field effects. Principal component analysis (PCA) of the transcriptome expression demonstrated clear separation between NU/LGIN and UC samples (Figure 4A). Some of the HGIN samples coclustered with NU/LGIN samples, whereas others coclustered with UC samples. The volcano plots in Figure 4B show differentially expressed genes in three groups of mucosal samples corresponding to NU/LGIN, HGIN, and UC as compared with control samples.

Overall, 20,795 genes were differentially expressed in at least one map sample when compared with that in NU samples obtained from patients with urothelial neoplasia. We then identified 1,274 abnormally expressed genes (502 upregulated and 772 downregulated), which demonstrated continuous dysregulation at the transition involving mucosal field effects (NU/LGIN) and progressing through HGIN to UC, referred to as monotonically altered. We observed two major waves of gene expression changes in the progression of neoplasia from field effects to invasive carcinoma (supplementary material, Figure S5). The first wave of these changes (*n* = 354 genes: 212 upregulated, 142 downregulated) contained abnormally expressed genes in samples of NU/LGIN whose expression became abnormal with the development of HGIN and progression to UC. The second wave of changes (*n* = 54 genes: 3 upregulated, 51 downregulated) consisted of genes that were abnormally expressed at the transition from NU/LGIN to HGIN and UC. The genes in the first wave had aberrant expression patterns in the early phases of bladder carcinogenesis, forming upregulated or downregulated plaques involving large areas of bladder mucosa extending to mucosal areas with no or minimal microscopic changes corresponding to NU/LGIN.

In examining these two groups of genes, we focused on the 10 most upregulated and downregulated genes, which had monotonically abnormal expression patterns in areas of mucosa corresponding to NU/LGIN through HGIN to UC (i.e. representing early incipient changes in bladder carcinogenesis) (Figure 4C,D). The recurring theme among the top 10 upregulated genes was upregulation of KRT6, KRT14, and KRT16, signifying activation of a basal undifferentiated phenotype. Complementary overexpression of the CXCL8 chemokine ligand and IL1R2 indicated early dysregulation of the immune microenvironment. Overexpression of the G0S2 switch protein FBXO5 as well as the transcription factor (TF) E2F1 is an early sign of cell‐cycle dysregulation. Downregulated genes included uroplakin *UPK3B*, a component of apical plaques of terminally differentiated urothelium, further signifying early dysregulation of the urothelial differentiation program in mucosal field effects initiating bladder carcinogenesis. Other downregulated genes included *PAX8*, *PIK3C2G*, *TFAP2B*, and *FOXA1*, representing a dysregulated signal transduction program. Kyoto Encyclopedia of Genes and Genomes (KEGG) analysis identified 31 monotonically dysregulated pathways, including carcinogenesis, interleukin and nuclear factor‐κB signaling, and fatty acid degradation (Figure 4E).

We also analyzed the expression patterns for previously identified luminal/basal markers; epithelial‐mesenchymal transition (EMT) complemented by activation of urothelial, neural, and mesenchymal regulons; and immune infiltrates (supplementary material, Figures S6A–F, S7A–D). The upregulation of basal markers with negative basal to luminal transition (BLT) scores was already evident in the mucosal field effect corresponding to samples classified as NU/LGIN and continued throughout progression of the disease through HGIN to invasive UC (supplementary material, Figure S6A–D). This confirmed our prior observations that the intrinsic molecular subtypes of bladder cancer are determined *de novo* and are consistent with animal model data implicating that the luminal and basal subtypes of urothelial cancer have distinct cells of origin [27, 28]. In this particular case, it confirmed that the basal molecular subtype of invasive UC originated from basal field effects.

Because EMT plays a major role in the development of many human epithelial cancers, including those that originate in the urothelium, we assessed its role in the evolution of bladder cancer from mucosal field effects. The core of the complex EMT circuitry consists of TGFB1, p53, and p63, with downstream targets representing members of the SNAIL, TWIST, ZEB, and FOX families, which downregulate E‐cadherin (CDH1) and other adhesion molecules, including claudin‐1 and tight junction protein 1 [29]. We previously found that EMT plays a major role in the development of basal bladder cancer and its progression to highly aggressive variants, such as sarcomatoid and small‐cell cancers [30, 31]. Therefore, we analyzed the involvement of EMT in the evolution of bladder cancer from mucosal field effects. Consistent with our prior observations, activation of permissive EMT components, such as upregulation of TGFB1, p53, and p63 target genes, was evident in field effects (supplementary material, Figure S6C). Widespread activation of EMT combined with negative EMT scores was associated with progression to invasive bladder cancer and was a late effect (supplementary material, Figure S6D). Progressive activation of EMT was in synchrony with dysregulation of the urothelial differentiation program disclosed by analysis of regulons. Loss of the activity of nearly half of the urothelial regulons was already evident in mucosal field effects corresponding to NU/LGIN and was accompanied by activation of mesenchymal regulons (supplementary material, Figure S6E,F).

We hypothesized that alterations of the immune microenvironment may play a role in the early incipient phases of bladder carcinogenesis and analyzed immune‐related genes in the evolution of bladder cancer from field effects. We previously observed that tumors developing along the luminal track were depleted from immune infiltration [3]. In contrast, tumors developing along the basal track had increased immune signatures. Consistent with this observation, an increased immune signature in our basal cancer was already evident in mucosal field effects (supplementary material, Figure S7A–D), but was associated with the expression signature for T‐cell exhaustion (supplementary material, Figure S8A,B).

Selected results of the mRNA‐based analysis were validated by immunohistochemistry, which confirmed the activation of the basal phenotype in mucosal field effects in the samples corresponding to NU/LGIN with the development of full basal phenotype in progression to HGIN and UC samples (supplementary material, Figure S9A). We also confirmed the presence of prominent T‐ (CD3/8‐positive) cell infiltrate in mucosal field effects (samples corresponding to NU/LGIN) and in the progression to HGIN and UC (supplementary material, Figure S9B), as well as the retention of all microsatellite instability proteins (MLH1, MSH2, MSH6, and PMS2) (supplementary material, Figure S9C).

### Proteomic profile of bladder cancer evolution from field effects

**Figure 5:**
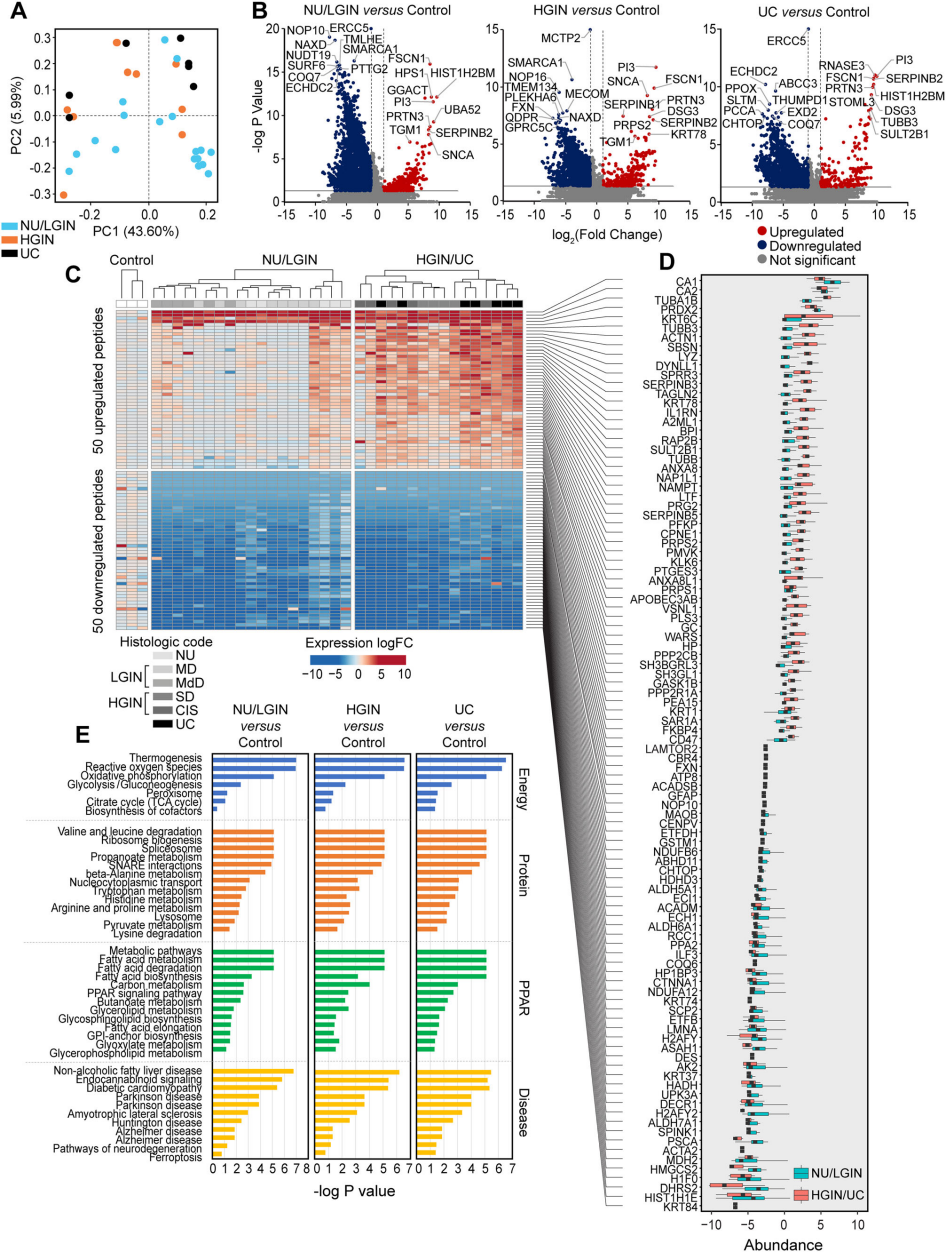
**Proteomic profile of bladder cancer evolution from mucosal field effects**. (A) PCA of protein expression data for all mucosal samples. (B) Volcano plots of all annotated proteins comparing log2 fold changes with −log *p* values in normal urethelium (NU)/low‐grade intraepithelial neoplasia (LGIN), high‐grade intraepithelial neoplasia (HGIN), and urothelial carcinoma (UC) samples versus those in control samples. (C) Heatmap of the top 50 upregulated and top 50 downregulated proteins in mucosal samples. (D) Boxplot of the top 50 upregulated and top 50 downregulated proteins in C depicting whole changes in NU/LGIN and HGIN/UC samples compared with controls. (E) Monotonically dysregulated KEGG protein pathways showing −log *p* values for the comparison of NU/LGIN, HGIN, and UC samples with control samples.

Proteomic profiling of 38 geographically mapped mucosal samples from a cystectomy identified 8,475 distinctive proteins, which are listed in the supplementary material, Table S8. Heatmaps of their expression patterns are shown in the supplementary material, Figure S10A,B. The proteome coverage for the individual mucosal samples ranged from 155 to 7,223 proteins (supplementary material, Figure S11A). We selected the samples from mucosal areas with more than 2,500 identified proteins for downstream analysis and grouped them as NU/LGIN, HGIN, and UC samples. We used the proteins extracted from normal ureters in nephrectomy samples from patients without urothelial neoplasia as references. The proteome abundance distribution was consistent across 38 selected mucosal samples, as shown in the supplementary material, Figure S11B. The number of gene products and peptide spectrum matches in individual mucosal samples and the rank order of normalized proteins are shown in the supplementary material, Figure S11C,D. PCA of the proteome composition of the samples demonstrated a clear separation of NU/LGIN versus HGIN and UC samples (Figure 5A). The HGIN and UC samples clustered together. Gene Ontology analysis of cellular components demonstrated that we recovered about 50% of the reported total number of proteins in each cellular compartment (supplementary material, Figure S11E). The volcano plots in Figure 5B show differentially expressed proteins in three groups of mucosal samples corresponding to NU/LGIN, HGIN, and UC, as compared with control samples.

In our protein analysis, we focused on the monotonic changes in protein expression that paralleled the progression of neoplasia from NU/LGIN through HGIN to UC. The total numbers of proteins with monotonic upregulation or downregulation in this progression were 2,343, 2,413, and 2,504 for NU/LGIN, HGIN, and UC, respectively, which we analyzed using the iPathwayGuide program. The top 50 monotonically upregulated and downregulated proteins are shown in Figure 5C,D. By using this approach, we identified 42 dysregulated protein pathways in the mucosal field effects corresponding to NU/LGIN that continued to be progressively dysregulated in the evolution of neoplasia through HGIN to UC (supplementary material, Table S9). The dysregulated pathways converging upon energy and protein homeostasis were complemented by tissue differentiation program defects (Figure 5E). The downregulated energy pathways included oxidative phosphorylation; valine, leucine, and isoleucine degradation; the citric acid cycle; and thermogenesis. The protein homeostasis defects included dysregulated nucleocytoplasmic transport, spliceosome, ribosome biogenesis, and peroxisome. The signature dysregulated pathway involved in tissue differentiation, lipid metabolism, and protein degradation is PPAR signaling. The dysregulation of PPAR signaling identified by the proteomic analysis was in synchrony with the downregulation of urothelial regulons including PPAR identified by RNASeq (supplementary material, Figure S6E,F).

When we compared protein expressions with RNASeq data 486 proteins with dysregulation evolving in the same direction as their corresponding mRNA were identified (supplementary material, Figure S12A). The top proteins from this group showed upregulation of multiple keratins such as KRT6, KRT14, and KRT17 associated with the activation of basal phenotype already evident in the mucosal field effects, i.e. corresponding to areas of bladder mucosa with microscopic features NU/LGIN (supplementary material, Figure S12B).

### Metabolomic analysis of bladder cancer evolution from field effects

Targeted metabolomic profiling of 38 geographically mapped mucosal samples from a cystectomy sample using positive and negative ionization modes (electrospray ionization) identified 91 metabolites (supplementary material, Table S10). Subsequently, we normalized the peak areas of metabolites using internal standards for further analysis. PCA demonstrated the clustering distinction among the samples from the NU/LGIN, HGIN, and UC groups and revealed preferential clustering of HGIN and UC samples (supplementary material, Figure S13A). Differentially expressed metabolites visualized on the PCA plot represented their log‐fold‐change values, with significantly upregulated and downregulated metabolites highlighted. The differentially expressed metabolites in the mucosal samples as compared with those in control samples and in comparisons of HGIN/UC and NU/LGIN samples are shown in the volcano plots in the supplementary material, Figure S13B. The total number of measured metabolites and their expression patterns in individual mucosal samples classified as NU/LGIN and HGIN/UC are shown in the heatmap and boxplot in the supplementary material, Figures S13C and S13D, respectively. The top five monotonically upregulated metabolites were glycerol‐3‐phosphate, betaine, farnesyl‐PP, taurine, and lactate, whereas the top five monotonically downregulated proteins were 5‐CMP, homocysteine, cystathionine, dCMP, and ADP.

The elevated glycerol‐3‐phosphate levels are linked to increased lipid biosynthesis and Warburg effect [32, 33, 34]. The increased levels of betaine may reflect a greater demand for histone methylation and chromatin remodeling of cells undergoing transformation [35]. On the other hand, higher levels of farnesyl‐pyrophosphate indicate activation of the mevalonate pathway playing an important role in the activation of Ras and Rho GTPase signaling, evident already in early field effects [36, 37]. Downregulation of homocysteine, cystathionine, dCMP, and ADP are indicative of compensatory effects to maintain oxidative stress resulting from mitochondrial dysfunction [38, 39, 40, 41, 42, 43]. These findings collectively suggest that mitochondrial dysfunction in mucosal field effects drive bladder carcinogenesis by providing metabolic adaptations that promote lipid synthesis, redox balance, and oncogenic signaling while supporting the energy needs of cells undergoing malignant transformation.

Furthermore, we conducted pathway analysis to compare (1) NU/LGIN and control, (2) HGIN and control, and (3) UC and control samples (supplementary material, Figure S13E). The top five monotonically enriched pathways were transfer RNA charging, citrulline biosynthesis, the superpathway of citrulline metabolism, arginine degradation VI, and proline biosynthesis II (from arginine). Analysis of enrichment scores (ESs) for metabolites in individual mucosal samples using a single‐sample gene set enrichment analysis (GSEA) identified 74 monotonically dysregulated pathways (supplementary material, Table S11). The top five dysregulated pathways were glycerolipid metabolism, steroid biosynthesis, terpenoid backbone biosynthesis, taurine and hypotaurine metabolism, and cAMP signaling. The top five downregulated pathways were platelet activation, cysteine and methionine metabolism, fatty acid elongation, fatty acid biosynthesis, and fatty acid degradation. Of note, elevation of lactate and activation of glycolysis were already evident in microscopically normal‐appearing areas of bladder mucosa, implicating the emergence of the Warburg phenotype in the field effects. Taken together, these findings demonstrated complex alterations of glucolipid energy‐related metabolism in field effects dysregulating the mucosal microenvironment in the incipient phases of bladder carcinogenesis.

### Integrated analysis of bladder cancer evolution from field effects

**Figure 6:**
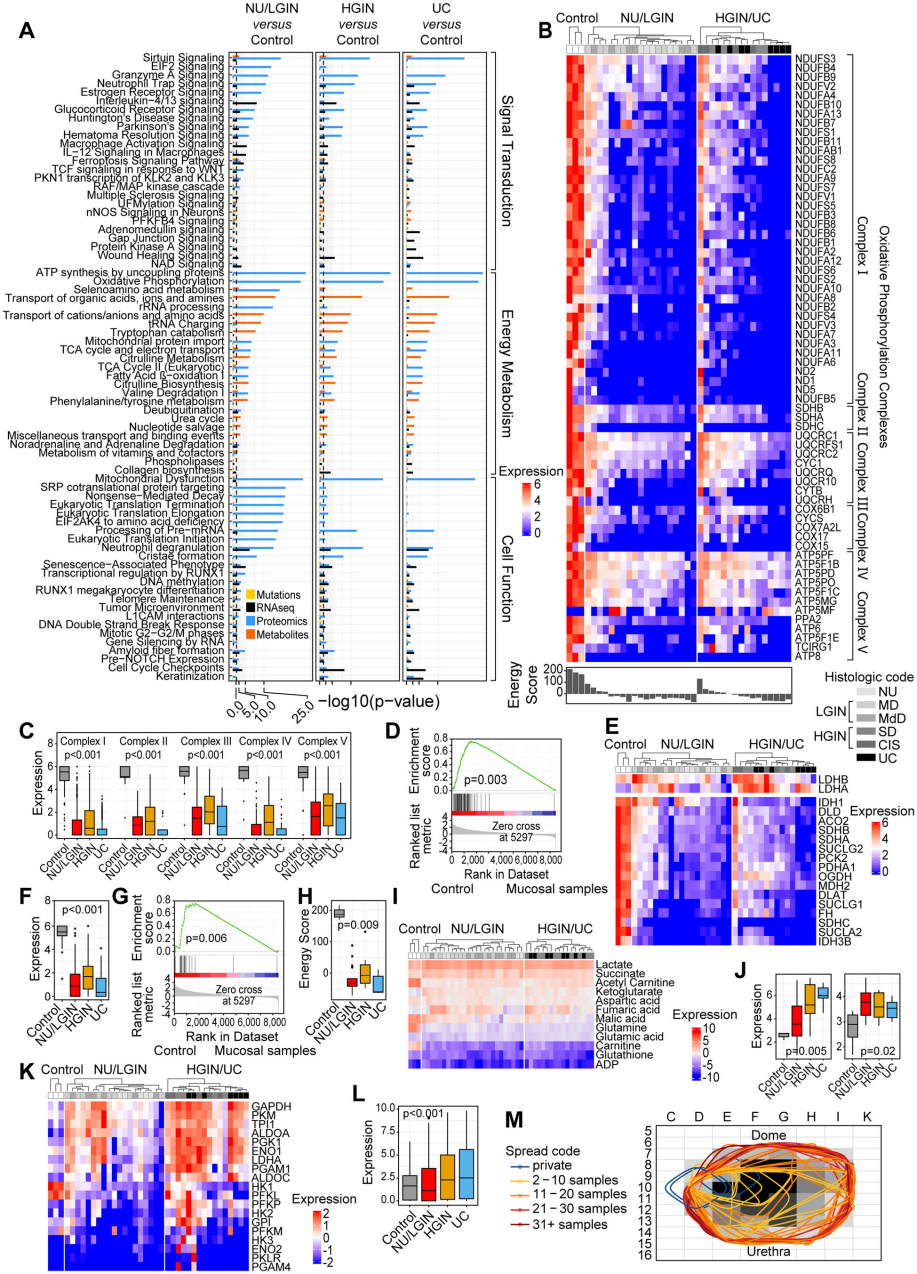
Interactive analysis of molecular pathways in bladder cancer development from field effects. (A) Combined analysis of monotonically dysregulated pathways in normal urethelium (NU)/low‐grade intraepithelial neoplasia (LGIN), high‐grade intraepithelial neoplasia (HGIN), and urothelial carcinoma (UC) (one‐sided Fisher's exact test *p* value). (B) Proteomic expression levels for enzymes in mitochondrial oxidative phosphorylation complexes. Enlarged views of panels A and B are available in the supplementary material, Figure S16. (C) Boxplot of the expression levels for enzymes in oxidative phosphorylation complexes in three groups of samples corresponding to NU/LGIN, HGIN, and UC compared with control urothelium. (D) GSEA of protein expression levels in oxidative phosphorylation complexes in the mucosal samples compared with control urothelium. (E) Expression levels for enzymes involved in the citric acid cycle. (F) Boxplot of expression levels for enzymes involved in the citric acid cycle in three groups of samples corresponding to NU/LGIN, HGIN, and UC compared with control urothelium. (G) GSEA of expression levels for proteins involved in the citric acid cycle in the mucosal samples compared with control urothelium. (H) Boxplot of the energy scores in three groups of samples corresponding to NU/LGIN, HGIN, and UC compared with control urothelium. (I) Expression levels for metabolites involved in the citric acid cycle. (J) Boxplot of lactate dehydrogenase A (left) and lactic acid (right) levels in three groups of samples corresponding to NU/LGIN, HGIN, and UC compared with control urothelium. (K) Expression levels for enzymes involved in glycolysis. (L) Boxplot of the expression levels for glycolysis enzymes in three groups of samples corresponding to NU/LGIN, HGIN, and UC compared with control urothelium. (M) Spatial distribution of the downregulated enzymes involved in mitochondrial oxidative phosphorylation in B superimposed on a histologic map of a cystectomy.

To identify pathways involved in the development of bladder cancer, we used β and γ mutations complemented with genome‐wide expression levels of monotonically altered genes combined with proteomic and metabolomic alterations. This integrated analysis identified 73 canonical pathways in NU/LGIN samples that were continuously altered in the progression to HGIN and UC (Figure 6A). These pathways could be classified into three major groups involving signal transduction, energy metabolism, and various cellular functions. Among these pathways, dysregulation of mitochondrial energy metabolism appeared to be the dominant change driving the progression of neoplasia from mucosal field effects to invasive cancer. This prompted a more detailed analysis that identified widespread dysregulation of oxidative phosphorylation with downregulation of enzymatic components of all five complexes combined with downregulation of the citric acid enzymes (Figure 6B–G). We assessed the oxidative energy metabolism using the energy score, which demonstrated its dramatic downregulation in mucosal field effects (Figure 6H). Metabolomic changes resulted in significant downregulation of the citric acid cycle, exemplified by decreased glutamine, acetylcarnitine, and carnitine with increased α‐ketoglutarate (Figure 6I). Downregulation of oxidative energy metabolism was accompanied by upregulation of glycolysis with an anaerobic switch, exemplified by increased levels of lactate and lactate dehydrogenase A (Figure 6J−L). These complex alterations of energy metabolism were evident in early mucosal field effects involving microscopically normal‐appearing urothelium and continued to be progressively dysregulated in the progression of neoplasia through *in situ* intraurothelial preneoplastic conditions to invasive cancer. An example of plaque‐like downregulation of mitochondrial oxidative phosphorylation enzymes in almost the entire bladder mucosa corresponding to an early field type change is shown in Figure 6M.

We validated the downregulation of oxidative phosphorylation identified in a single cystectomy specimen in the TCGA cohort (*n* = 408) of bladder cancer (supplementary material, Figure S14A–E). It showed that widespread downregulation of mitochondrial oxidative phosphorylation is a common feature in bladder cancer. It is enriched in basal and double‐negative molecular subsets of the disease, with downregulation in over 50% of the cases in these subtypes.

## Discussion

This study provided proof of principle regarding the validity and feasibility of multiplatform mutational, proteomic, and metabolomic analysis on the whole‐organ scale to infer the molecular profile of bladder cancer development from mucosal field effects. It provided evidence for time modeling of the mutational landscape in the context of complex alterations involving protein homeostasis and the tissue differentiation program combined with dysregulation of glucolipid energy‐related metabolism. Proteomic profiling of bladder cancer tissue performed by others revealed subtype‐ and stage‐related differences converging upon glucolipid metabolism [44, 45, 46]. Whole‐organ spatial geographic analyses revealed that bladder carcinogenesis may develop innocuously over several decades and can be divided into dormant and progressive phases. The mutational landscape develops gradually via multiple waves corresponding to distinct successive clones of cells, which may develop along several distinct branches. The most common mutations are low‐frequency α mutations restricted to individual mucosal samples. They very likely represent the progeny of individual uroprogenitor cells. The α mutations occurred early and did so continuously over three decades. The β and γ mutations were associated with intramucosal clonal expansion. β mutations had low VAFs, typically occurring in less than 10% of cells. The emergence of clones with β mutations was associated with a transition to the progressive phase during the last 5 years of carcinogenesis. Finally, γ mutations appeared to be drivers of the progressive phase, emerging 2–3 years before the progression to invasive cancer.

We identified highly variable mutagenesis signatures in individual mucosal samples, but signatures 1 and 6 were dominant [47]. Moreover, the development of α, β, and γ mutations was driven by distinct mutational mechanisms. Similarly, the transition from the dormant to the progressive phase of carcinogenesis was associated with the involvement of different mutagenesis signatures, confirming that the progressive phase of bladder carcinogenesis developed through the activation of distinct mutational mechanisms.

These mutational landscape changes developed on a background of complex gene expression, proteomic and metabolomic dysregulation. Analysis of the RNA‐seq expression profile identified intricate dysregulation of the urothelial differentiation program with features of basalization evident already in the incipient field effects. These changes were associated with activation of EMT and loss of activities in nearly half of urothelial regulons combined with an altered mucosal immune microenvironment exhibiting features of T‐cell exhaustion. The proteome alterations consisted of defects in protein homeostasis, including dysregulated nucleocytoplasmic transport, spliceosome, ribosome biogenesis, and peroxisome. Notably, these changes were associated with altered urothelial differentiation controlled by PPAR and involved lipid metabolism and protein degradations [48, 49]. Proteome alterations were accompanied by equally complex dysregulation of glycerolipid metabolism, steroid biosynthesis, terpenoid backbone biosynthesis, taurine and hypotaurine metabolism, and cAMP signaling. The top dysregulated metabolomic pathways were cysteine and methionine metabolism, fatty acid elongation, fatty acid biosynthesis, and fatty acid degradation, including activated glycolysis, signifying complex alterations of glucolipid energy‐related metabolism in field effects, which complemented the dysregulated mucosal microenvironment in the incipient phases of bladder carcinogenesis.

Increased levels of lactic acid in mucosal samples indicate a shift toward anaerobic glycolysis, likely triggered by insufficient oxygen supply and mitochondrial dysfunction [50]. Elevated lactate production in the early phases of bladder carcinogenesis identified in the present study contributed to mucosal microenvironment acidification, which enhances cell survival, proliferation, and metastasis. High lactate levels have been linked with increased tumor aggressiveness and poor prognosis for other types of cancer [51]. Herein we show that this metabolic change is an early event in bladder carcinogenesis and can be found in microscopically NU adjacent to precursor lesions, such as severe dysplasia and carcinoma *in situ* (CIS). Collectively, these changes demonstrate that preneoplastic cells undergo metabolic reprogramming, relying predominantly on glucose and glutamine for energy production, while struggling to use fatty acids effectively. This metabolic state results in less efficient ATP generation and may be part of a broader cellular stress response. Researchers also observed such alterations with various nonneoplastic conditions involving mitochondrial dysfunction, including neurodegenerative diseases, cardiovascular disorders, and metabolic syndromes [52, 53, 54]. Our integrated multiplatform analysis identified multiple monotonically dysregulated pathways in mucosal field effects. It also disclosed that dysregulation of mitochondrial energy metabolism with widespread downregulation of oxidative phosphorylation, and the citric acid cycle with upregulation of anaerobic glycolysis, was a leading mechanism driving the progression of field effects through *in situ* preneoplastic conditions to invasive bladder cancer.

Whole‐organ mapping provides a convincing sequence of events within each platform. On the other hand, the timing of the relationship among the platforms is problematic, and clear articulation whether metabolic reprogramming precedes or follows mutational change is uncertain. However, the α mutations, which are heterogenous and restricted to smaller mucosal areas, are unlikely to cause a diffuse monotonic mucosal change. Therefore, the diffuse mucosal metabolic change, such as downregulation of mitochondrial oxidative phosphorylation, is likely related to the advent of β mutations followed by the γ mutations, which clonally expanded and appeared to be drivers of the progression to invasive disease.

This is a proof‐of‐principle study based on whole‐organ genomic (DNA and RNA), proteomic, and metabolomic whole‐organ mapping of a single cystectomy with bladder cancer, and it is not intended to reflect the general profile of bladder cancer evolution from mucosal field effects. Comparisons of our observations with those of the TCGA cohort showed that both the mutational signatures, as well as dysregulation of mitochondrial oxidative phosphorylation, are common features in bladder cancer. Therefore, even though this study is restricted to one cystectomy specimen, it represents an advancement of our knowledge on molecular mechanisms of bladder cancer development from urothelial mucosa. The use of normal urothelium from ureters of nephrectomies is a compromise related to its availability for reference purposes. However, we acknowledge that it is not identical to the normal urothelium of the bladder and this can be perceived as a limitation of our analytical strategy [55].

Our observations are in synchrony with work by others demonstrating that early events of carcinogenesis include complex metabolic alterations converging on glycolysis and oxidative phosphorylation, favoring early clonal expansion of cells with the Warburg phenotype [56, 57, 58, 59]. Of note, recent lipidomic profiling studies identified unique lipid signatures of bladder cancer associated with clinical stage and ethnicity [60, 61, 62].

## Author contributions statement

BC conceived, designed, and supervised the study and wrote the article. SL, JB, JGL and DC performed pregenomic laboratory experiments. SYJ performed proteomic analysis. NP performed metabolomic analysis. PK, RJ, KND, HC, YW and JC performed data analysis. NN and CD selected the patient and provided clinical data. CM, DM and RRB interpreted the data. CCG provided pathological classification. PW and MK designed and supervised the analytical protocols. All the authors participated in the data interpretation and critically revised and approved the final article.

## Supporting information


Supplementary materials and methods
**Figure S1.** Histograms of VAFs of α, β, and γ mutations in individual cystectomy samples
**Figure S2.** Mutational landscape of bladder cancer evolution from field effects after filtration for COSMIC mutation
**Figure S3.** Analysis of pathways affected by α, β, and γ mutations after filtration for COSMIC mutations
**Figure S4.** Mutational signature of bladder cancer evolution from mucosal field effects
**Figure S5.** Expression profile for monotonically dysregulated genes identified via RNA‐seq of all mucosal samples from a cystectomy sample
**Figure S6.** Expression profiles for selected genes identified via RNA‐seq of all mucosal samples from a cystectomy sample
**Figure S7.** Immune landscape of bladder cancer evolution from field effects
**Figure S8.** T‐cell exhaustion signatures in progression to bladder cancer from mucosal field effects
**Figure S9.** Immunohistochemical validation of the basal field effects, immune infiltration, and retention of microsatellite stability gene
**Figure S10.** Expression patterns for proteins identified by sequencing of all mucosal samples from a cystectomy sample
**Figure S11.** Proteome profiling of a whole‐organ map of a cystectomy sample obtained from a patient with bladder cancer
**Figure S12.** Heatmap of proteins showing alterations in the same direction as their respective mRNA
**Figure S13.** Metabolomic profile of bladder cancer evolution from mucosal field effects
**Figure S14.** Expression pattern of mRNA encoding enzymes involved in mitochondrial oxidative phosphorylation in the TCGA cohort (*n* = 408)
**Figure S15.** Enlarged views of panels B and D from Figure 2
**Figure S16.** Enlarged views of panels A and B from Figure 6
**Table S1.** Summary of mutations identified in the cystectomy specimen
**Table S2.** List of α mutations and their VAFs in individual mucosal samples of the cystectomy
**Table S3.** List of β mutations and their VAFs in individual mucosal samples of the cystectomy
**Table S4.** List of γ mutations and their VAFs in individual mucosal samples of the cystectomy
**Table S5.** List of α mutations filtered for COSMIC mutations and their VAFs in individual mucosal samples of the cystectomy
**Table S6.** List of β mutations and their VAFs in individual mucosal samples of the cystectomy
**Table S7.** List of γ mutations filtered for COSMIC mutations and their VAFs in individual mucosal samples of the cystectomy
**Table S8.** Expression levels of 8,475 proteins in individual mucosal samples
**Table S9.** Monotonically dysregulated proteomic pathways in progression of bladder cancer from field effects
**Table S10.** Expression levels of metabolites identified by targeted metabolomics in individual mucosal samples
**Table S11.** Activation score of KEGG metabolomic pathways in individual mucosal samples

## Data Availability

The whole‐exome DNA/RNA sequencing data, both raw and analyzed, were deposited in the Sequence Read Archive (PRJNA1065919). The MS data for proteome profiling were deposited via the MASSIVE repository (MSV000085220) to the ProteomeXchange Consortium (http://proteomecentral.proteomexchange.org) with the dataset identifier (PXD018341).
