# Supplementary material for: Dysregulated mitochondrial energy metabolism drives the progression of mucosal field effects to invasive bladder cancer
Source: J Pathol. 2025 Sep 25;267(3):329–46. doi: 10.1002/path.6474 (PMC12531120; doi:10.1002/path.6474)
Supplement: Supplementary file 1 — Supplementary materials and methods Figure S1. Histograms of VAFs of α, β, and γ mutations in individual cystectomy samples Figure S2. Mutational landscape of bladder cancer evolution from field effects after filtration for COSMIC mutation Figure S3. Analysis of pathways affected by α, β, and γ mutations after filtration for COSMIC mutations Figure S4. Mutational signature of bladder cancer evolution from mucosal field effects Figure S5. Expression profile for monotonically dysregulated genes identified via RNA‐seq of all mucosal samples from a cystectomy sample Figure S6. Expression profiles for selected genes identified via RNA‐seq of all mucosal samples from a cystectomy sample Figure S7. Immune landscape of bladder cancer evolution from field effects Figure S8. T‐cell exhaustion signatures in progression to bladder cancer from mucosal field effects Figure S9. Immunohistochemical validation of the basal field effects, immune infiltration, and retention of microsatellite stability gene Figure S10. Expression patterns for proteins identified by sequencing of all mucosal samples from a cystectomy sample Figure S11. Proteome profiling of a whole‐organ map of a cystectomy sample obtained from a patient with bladder cancer Figure S12. Heatmap of proteins showing alterations in the same direction as their respective mRNA Figure S13. Metabolomic profile of bladder cancer evolution from mucosal field effects Figure S14. Expression pattern of mRNA encoding enzymes involved in mitochondrial oxidative phosphorylation in the TCGA cohort (n = 408) Figure S15. Enlarged views of panels B and D from Figure 2 Figure S16. Enlarged views of panels A and B from Figure 6 Table S1. Summary of mutations identified in the cystectomy specimen Table S2. List of α mutations and their VAFs in individual mucosal samples of the cystectomy Table S3. List of β mutations and their VAFs in individual mucosal samples of the cystectomy Table S4. List of γ mutations and their VAFs in individual mucos [file PATH-267-329-s001.zip › path6474-sup-0001-SuppMatMethFiguresS1-S16TablesS1-S9.docx]

**Dysregulated mitochondrial energy metabolism drives the progression of mucosal field effects to invasive bladder cancer**

S Lee, SY Jung, P Kuś *et al. J Pathol* <https://doi.org/10.1002/path.6474>

**Supplementary materials and methods**

**Supplementary Figures S1–S14**

**Supplementary Tables S1 and S9**

**Supplementary Tables S2–S8, S10, and S11 (provided in separate Excel files)**

Reference numbers refer to the main text list.

**Supplementary materials and methods**

**Experimental model and subject details**

In brief, preparation of the cystectomy sample for whole-organ histologic mapping combined with DNA/RNA, protein, and metabolite extraction followed the steps illustrated in Figure 1A–J. The cystectomy sample was opened longitudinally along the anterior wall of the bladder and pinned down to a paraffin block. A mapping grid was then superimposed and pressed down over the bladder mucosa with mechanical screws. The mapping grid had sealed wells that separated mucosal areas into 1 × 2-cm (2 cm^2^) rectangles. Phosphate-buffered saline (1 ml) was poured into each well, the surface urothelium was scraped, and the fluid was collected into Eppendorf tubes. The urothelial cells were spun down and resuspended in phosphate-buffered saline. In areas of mucosa containing grossly recognizable tumor, the tumor tissue was collected via direct dissection from the bladder wall, cut into small pieces, and processed as described above. The final 600-μl cell suspensions were divided into three parts, with two parts kept frozen in phosphate-buffered saline with 10% dimethyl sulfoxide until processed for protein and metabolite extraction. For DNA/RNA extraction, the cell suspensions were treated with TRIzol reagents after spinning down and kept frozen. The mapping grid was removed from the surface of the bladder, which was then fixed in formalin overnight. The grooves at the bottom of the mapping grid left permanent impressions on the bladder surface and preserved the urothelium for microscopic inspection and histologic mapping of the entire bladder mucosa. Paraffin-embedded sections of bladder mucosa corresponding to mapping grooves were collected and were stained with hematoxylin and eosin to evaluate the distribution of microscopically normal urothelium (NU), *in situ* precursor lesions, and urothelial carcinoma (UC). Intraurothelial lesions were dichotomized into low-grade intraepithelial neoplasia (LGIN) and high-grade intraepithelial neoplasia (HGIN) categories as previously described [10-12]. Samples with tumor tissue were classified according to the two-tier histologic grading system of the World Health Organization (low and high grade) [63]. The growth patterns of papillary *versus* solid and the depth of invasion were recorded. Levels of invasion were defined according to the TNM staging system [64,65].

Two steps were used for quality check controls, which consisted of overall assessment of the cystectomy and determining the quality of the final DNA/RNA and protein preparations for genomic and proteomic profiling. In the first step, the cystectomy sample was assessed in terms of representation of the whole spectrum of the *in situ* precursor lesions and tumor samples as well as the purity of the urothelial and tumor cell preparations. In the second step, the quality of the DNA/RNA preparations was verified using a NanoDrop spectrophotometer (NanoDrop

Technologies Inc., Wilmington, DE, USA), Bioanalyzer system (Agilent, Santa Clara, CA, USA), and Qubit fluorometer (Thermo Fisher Scientiﬁc, Waltham, MA, USA).

**Whole-exome sequencing and data analysis**

The initial alignment of reads to the GRCh38 reference genome was performed with the BWA-MEM algorithm (v0.7.12, <http://bio-bwa.sourceforge.net>, 06/08/2025). The Genome Analysis Toolkit (v3.4-46, <https://gatk.broadinstitute.org>, 06/08/2025) was used to generate realigned and recalibrated BAM files. The MuTect2 and Oncotator (v1.8.0.0, <https://gatk.broadinstitute.org/hc/en-us>, 06/08/2025) tools were used to identify mutations.

**Germ line mutations analysis**

Whole-exome sequencing (WES) data from three normal control samples were preprocessed using BWA-MEM (v0.7.17**,** <http://bio-bwa.sourceforge.net>, 06/08/2025) to align reads to the GRCh38 reference genome. Picard (v2.27.4, <https://broadinstitute.github.io/picard>, 06/08/2025) was used to sort and convert file formats as well as to remove duplicate reads. The Genome Analysis Toolkit (GATK v3.7, <https://software.broadinstitute.org/gatk>, 06/08/2025) was employed to perform local realignment and base quality score recalibration, resulting in finalized BAM files.

Subsequently, GATK4 (v4.4.0.0, <https://gatk.broadinstitute.org>, 06/08/2025) was used to detect germline single nucleotide variants (SNVs). Intermediate GVCF files were generated for each sample and then combined into a single merged GVCF file across all samples. Variant Quality Score Recalibration (VQSR) was applied to assign well-calibrated probabilities to each variant call. A tranche sensitivity threshold of 99.0% was used to filter high-confidence variants.

**Mutational signatures**

Mutational fingerprints in quadratic programming were used to estimate a weight score for each mutational signature using data from the Sanger Institute database (https://cancer.sanger.ac.uk/cosmic/signatures) as previously described [3,4]. A matrix of canonical signatures with the mutational profile of a sample was used to compute the 30 × 1 vector for each of the canonical signatures’ relative contributions to the sample mutagenesis profile by computing the following optimization:

minH (WH – V)T(WH – V) such that hi ≥ 0 and Σi hi = 1,

in which H is the weight score, W is the mutational signature, and V is the mutational fingerprint.

The Kruskal–Wallis test was used to test the null hypothesis of no difference in weight scores among the groups of mucosal samples. Bootstrapping was applied to assessment of the contribution of mutational signatures in mucosal samples. Mutational fingerprints for each sample were resampled with replacement, and the weight scores were computed as described above 2,000 times. The one-sided empirical *p*-value was computed as the percentage of weight scores equal to or greater than the weight score in the resampling distribution.

**Phylogenetic analysis and modeling of bladder cancer evolution**

A phylogenetic tree was reconstructed by computing the Hamming distances of the mutations in mucosal samples using their matrixes of all nonsilent and silent mutations using the maximum parsimony algorithm [3]. In a graphical representation of a phylogenetic tree, each node corresponds to a population of cells, and the length of the edge connecting the nodes is proportional to the number of mutations. A branch represents a point in the evolution where two distinct populations emerge, whereas the length of the branch is proportional to the number of mutations that are unique for each population.

The time evolution of the mutational landscape in bladder cancer evolution from mucosal field effects was reconstructed by using a parsimonious time-continuous Markov branching process [14]. In brief, a mutation $j$ appears at the time $t_{0}^{j}$ in a progenitor cell of the urinary bladder urothelial lining and gives rise to a mutant clone. Mutant cells divide at rate $\lambda_{j}$ (1/year), and after division, one cell enters self-renewal, the other cell differentiates with probability $1-s_{j}$ or both cells enter self-renewal with probability $s_{j}$. As a consequence, the mutant clone grows exponentially as $\exp\left( \lambda_{j}s_{j}t \right)$, where $t$ is the age of the $j$-th mutant’s clone counted from $t_{0}^{j}$. The secondary clones expand, involving different areas of bladder mucosa at times $t_{i}^{j}, i\geq0$ modeled using a stochastic Poisson process with intensity $\nu$ (1/year) [66]. If the expected cell counts in the successive $j$-th mutant clones are denoted by$X_{i}^{j}\left( t \right),$ $i = 0, 1, 2,\ldots$, and the number of haploid genomes in normal uroprogenitor cells is denoted by $2N$, the corresponding VAFs $V_{i}^{j}\left( t \right)$ are defined as the ratios $V_{i}^{j}\left( t \right) = X_{i}^{j}\left( t \right)/(2N)$ and are computed as follows [14]:

$$E\left[ V_{i}^{j}\left( t \right) \right] = \exp\left( \lambda_{j}s_{j}t \right)\left( \frac{\nu_{j}}{\nu_{j}+\lambda_{j}s_{j}} \right)^{i}\int_{0}^{\left( \nu_{i}+\lambda_{j}s_{j} \right)t} \frac{u^{i-1}}{\left( i-1 \right)!}\exp\left( -u \right)du/(2N), i = 0, 1, 2,\ldots.$$

For any mutation $j$ of age $t_{j}$, the sequence of expectations $E\left[ V_{i}^{j}\left( t_{j} \right) \right], i = 0, 1, 2,\ldots,$ was computed to estimate the coefficients $a_{j} = \lambda s_{j}t_{j}$ and $b_{j} = \nu_{j}t_{j}$. With a cell division rate $\lambda_{j}$ and migration rate $v_{j}$, the parameter $b_{j}$ is the proxy for mutation age $t_{j}$, whereas the ratio $a_{j}/b_{j}$ is the proxy for selection coefficient $s_{j}$.The coefficient $c = 2N$ is a constant parameter representing an estimate of the number of uroprogenitor cells in the sampled area. The computations were performed for 10^2^-10^5^ uroprogenitor cells in the sampled mucosal area, which did not significantly change the time modeling results. The best fit was obtained with 5 × 10^3^ uroprogenitor cells, for which the data are presented.

The objective was reconstruction of the evolution of the mutational landscape from mucosal field effects to invasive cancer in the forward time by connecting the migration of urothelial cells to their proliferation rates. A parsimonious model assumes that the migration rate is proportional to the power $\sigma$ of the proliferation rate (i.e. $\nu_{j} = \nu_{0}\rho_{j}^{\sigma}$). Hence, the parameter $b_{j} = \nu_{j}t_{j}$ for mutant $j$ has the form $b_{j} = \nu_{0}{\rho_{j}^{\sigma}t}_{j},$ where $\rho_{j} = {\lambda_{j}s}_{j}$ (and $\nu_{0}$ is a reference migration rate), and has the form $a_{j} = t_{j}\rho_{j}$. Solving these two equations for $\rho_{j}$ and $t_{j}$ provides estimates for the proliferation rate (proxy for the selection coefficient) and mutation age of mutant $j$as follows:

$$\rho_{j} = \left( {b_{j}}/{(a_{j}\nu_{0})} \right)^{1/{(\sigma-1)}}, t_{j} = a_{j}^{1+1/{(\sigma-1)}}\left( {\nu_{0}}/{b_{j}} \right)^{1/{(\sigma-1)}}$$

A series of extensive parametric studies was carried out, demonstrating that estimates corresponding to high values for parameter *σ*, such as σ = 6, fit the chronology of different mutation classes (α, β, and γ), which is consistent with biological and clinical intuition. A fitting algorithm with optimization programs and the *fminsearch* and *fminbnd* functions in the MATLAB (Natick, MA, USA) programming language was used to estimate the sequence of mutations in tumor development [67-69]. The resulting time estimates are presented as bar diagrams representing the age of mutations and point charts for the corresponding selection coefficients.

**RNA-seq and data analysis**

Quality control for RNA was conducted using RSeQC [70] and FastQC software (v0.11.5, <https://www.bioinformatics.babraham.ac.uk/projects/fastqc>, 06/08/2025). Sequencing reads were aligned to the GRCh38 reference genome using STAR (v2.7.3a) [71] with GENCODE (release 32) transcript annotations [72]. Read counts for individual genes were obtained using featureCounts software in the Subread package [73]. On average, 27 million reads per sample associated with more than 58,000 unique genes, both coding and noncoding, were obtained.

From this set of genes, 44,000 with more than 10 reads in at least one sample were selected and used in the subsequent analyses. Genes differentially expressed in specific sample groups were identified using DESeq2 software (v1.26.0) [15] with the Wald test by using a design formula that included batch effect correction. Benjamini–Hochberg correction was used for multiple testing in all instances [13].

The normalized read counts were transformed into log2 ratios of the signal of NU/LGIN, HGIN, and UC samples to the average signal obtained from the normal control samples. The resulting log fold-change values were used for the detection of monotonically dysregulated genes in three groups of mucosal samples, which followed the progression pattern of neoplasia from field effects (NU/LGIN) through HGIN to UC as follows. (1) The genes in the first group were dysregulated in early field effects (NU/LGIN), and their dysregulation continued in the same direction with progression to HGIN and UC. They were identified according to their different expression levels in at least 80% of all samples, and their expression levels were upregulated or downregulated by a log fold-change of at least 1.5; (2) The genes in the second group were dysregulated in parallel with disease progression to HGIN, and their dysregulation continued in the same direction as their dysregulation in progression to UC. They were identified according to their dysregulation in 80% of HGIN/UC samples, with expression levels altered by a log fold-change of at least 1.5; (3) The genes in the third group were identified according to their dysregulation, with a log fold-change of at least 1.5 in UC samples only.

For assessment of the luminal and basal phenotypes, the expression levels for 28 luminal and 20 basal marker genes that were identified previously were used. For quantitative assessment of these two molecular subtypes of bladder cancer, the previously developed BLT score was used [74]. In brief, for assessment of the luminal phenotype, the 14 luminal markers in the original classifier were used [75,76]. To increase the power of analyses, these markers were complemented by 14 PPARγ target genes previously shown to be significantly enriched in luminal cancers [75,76]. Similarly, for assessment of the basal phenotype, the nine basal markers from the original classifier were used, and they were complemented by an additional 11 p63 target genes shown to be significantly enriched in basal cancers [75,76]. Linear discriminant analysis was performed to determine the power of individual markers in identifying molecular subtypes of bladder cancer [77]. The unidimensional BLT score was defined as $\sum W_{i}*E_{i}$, where $W_{i}$ is the negative coefficient of the linear discriminant and *E*_i_ is the expression of marker genes. Next, least absolute shrinkage and selection operator analysis was used to select the 16 best luminal and 12 best basal markers to combat multicollinearity [78]. Specifically, least absolute shrinkage and a selection operator were applied to the L1 parameter to constrain the sum of the absolute values for the model parameters. In this process, 28 genes with nonzero coefficients after the regularization process were selected for calculation of the BLT score. A cohort from The Cancer Genome Atlas was used as a training set to build a linear discriminant analysis model with the 28 selected genes.

To assess the EMT status in the evolution of bladder cancer from mucosal field effects, the expression levels for signature TFs in the SNAIL, TWIST, ZEB, FOX, SOX, and KLF families that are involved in the activation of EMT were analyzed. This analysis was complemented by analyses of the expression of homotypic adhesion molecules such as E-cadherin, claudin-1, and tight junction protein 1. To quantitatively assess the level of EMT, the EMT score was calculated based on a 76-gene expression signature reported by Byers *et al* [30,79]. For each sample, the score was calculated as a weighted sum of 76 gene expression levels using $\sum_{i = 1}^{76} w_{i}G_{ij}$, where $w_{i}$ is the correlation coefficient between the ith gene expression in the signature and that of E-cadherin and *G*_ij_ is the ith gene’s normalized expression in the jth sample. The scores were centered by subtracting the mean for all tumor samples so that the grand mean of the score was zero.

Regulon analysis was also performed to infer the relative activity of two sets of candidate TFs [80]. For a given TF, the set of its putative target genes is defined as a regulon. The first set of TFs was previously reported to be associated with bladder cancer and analyzed for the gene regulatory network: *FOXA1*, *RXRA*, *FGFR3*, *RXRB*, *ERBB3*, *AR*, *GATA3*, *ESR2*, *ERBB2*, *PPARG*, *RARA*, *FGFR1*, *PGR*, *RARB*, *TP63*, *ESR1*, *GATA6*, *FOXM1*, *KLF4*, *EGFR*, and *HIF1A* [9]. The second set of TFs consisted of those reported to be related to the mesenchymal phenotype and manually curated based on the literature, including *SNAI2*, *IRX3*, *HOXC6*, *TWIST1*, *TULP3*, *PRRX1*, *BCL11A*, *TFEC*, *ERG*, *PLAGL2*, *DACH2*, *POU2F1*, *MMP9*, *STAT3*, and *SMAD3*.

To construct the regulons for the TFs of interest, a method developed and implemented in the R package RTN1 was employed [80,81]. In addition, Ingenuity Pathway Analysis (Ingenuity Systems, Redwood City, CA, USA) was used to supplement the list of target genes for a TF if the number of target genes identified using the RTN method was too small to allow reliable downstream analyses.

Each regulon was analyzed using two-tailed GSEA [80,82]. This method first divided the set of target genes for each TF into positive and negative targets associated with the phenotype of interest (e.g. conventional UC *versus* squamous cell carcinoma) using Spearman’s correlation coefficient. The distribution of both the positive and negative targets was tested, producing ESs for each sample. The difference between positive and negative ESs resulted in the differential ES. For further analysis of the difference between the conventional UC and squamous cell carcinoma, the differential ES was determined for each sample. How the regulons were associated with bladder cancer subtypes was assessed by analyzing the differential ES using heatmaps and two-tailed GSEA.

To analyze immune gene expression signatures of bladder cancer evolution from field effects, dendrogram nodes corresponding to genes expressed in specific immune cell types were identified via DAVID Functional Annotation Clustering and Ingenuity Pathway Analysis (<https://davidbioinformatics.nih.gov>, 06/08/2025) [83]. The immune expression signature was quantitatively assessed by calculating the immune scores for the expression profiles for genes [30]. Specifically, each of the genes in the immune gene set was standardized to have a mean of 0 and variance of 1. Next, for each sample, the weighted standardized expression values were summed for all of the genes, with upregulated genes having weights of +1 and downregulated genes having weights of −1. The resulting immune scores were then grouped and visualized using boxplots for samples belonging to each of four groups (control, NU/LGIN, HGIN, and UC). A T-cell exhaustion score was similarly constructed. The significance of the differences among the four groups was assessed using the Kruskal–Wallis test.

**Mass spectrometry-based proteome profiling**

Frozen tissue was resuspended and lysed in 50 mM ammonium bicarbonate, 1 mM CaCl_2_ via sonication for 3 min, and 50 μg of tissue lysate were digested using 1 μg of trypsin for 12 h at 37 °C. The digested peptide concentration was measured using a colorimetric peptide assay kit (Thermo Fisher Scientific, Waltham, MA, USA), and 25 μg of peptides was separated in a homemade high-pH reverse-phase C18 column in a pipet tip. Peptides were eluted and separated into 15 fractions using a stepwise acetonitrile gradient (2–30%, with 2% increments) at pH 10 and then combined into five fractions (2+12+12, 4+14+24, 6+16+26, 8+18+28, and 10+20+30) and vacuum-dried. The dried peptide samples were analyzed using an Orbitrap Fusion mass spectrometer (Thermo Fisher Scientific) coupled with an Easy-nLC 1000 nanoflow liquid chromatography system (Thermo Fisher Scientific). An in-house trap column (2 cm × 100 μm i.d.) and a 5 cm × 150-μm capillary separation column packed with 1.9 μm Reprosil-Pur Basic C18 beads (Dr. Maisch, Baden-Württemberg, Germany) were used for high-performance nano-liquid chromatography separation in a discontinuous gradient of 4–26% acetonitrile with 0.1% formic acid at a flow rate of 800 nl/min. Mass spectrometry (MS) was performed in data-dependent mode, acquiring fragmentation spectra for the 30 strongest ions under the control of Xcalibur software (v4.1; Thermo Fisher Scientific). The parental ion was acquired using the Thermo Scientific™ Orbitrap Fusion™ Tribrid™ mass spectrometer (Thermo Fisher Scientific) with a full MS range of 300–1,400 m/z at a resolution of 120,000. The higher energy collisional dissociation–fragmented MS/MS spectrum was acquired using an ion trap in rapid scan mode. The target-decoy Human RefSeq database (release January 21, 2020, containing 80,872 entries) was searched for the MS/MS spectra using the Proteome Discoverer 2.1 interface (Thermo Fisher Scientific) with the Mascot algorithm (v2.4, Matrix Science, London, UK). A precursor mass tolerance of 20 ppm and fragment mass tolerance of 0.5 Da were allowed. Two maximum missed cleavage and dynamic modifications of acetylation of N-term and oxidation of methionine also were allowed. Assigned peptides were filtered with a 1% FDR using Percolator validation based on the q-value. The peptide spectrum matches output from PD2.1 was used to group peptides at the gene level using the in-house gpGrouper algorithm [84]. This algorithm uses a universal peptide grouping logic to accurately allocate and provide MS1-based quantification across multiple gene products. Gene-protein product quantification was performed using label-free intensity-based absolute quantification (iBAQ) and then normalized to the fraction of total (a fraction of the total protein iBAQ amount per experiment). Fraction of total was defined as an individual protein's iBAQ divided by the total iBAQ for all identified proteins within one experiment.

The missing values in proteome recovery were replaced with half of the minimally detected values in the entire dataset. Following log2 transformation of this dataset, differential analysis (*t*-test) was performed, comparing one specific group with all the remaining samples combined. This step was repeated for all experimental groups [85]. Any protein was deemed to have significantly altered expression if it had a *p*-value less than 0.05 and a change greater than 1.5-fold. The selected gene-protein products were analyzed using iPathwayGuide (Advaita Bioinformatics, Raleigh, NC, USA), a systems biology-based approach to pathway-level analysis [86]. Unsupervised hierarchical clustering of normalized protein expression values was performed using software with the R language, and the results were visualized using the R package ComplexHeatmap. Pearson's correlation, mean centering, and average linkage were applied in all clustering applications.

Expression values for proteins within each mitochondrial oxidative phosphorylation complex, the citric acid cycle, and glycolysis in samples belonging to each of the four histologic groups (NU/LGIN, HGIN, and UC compared with control urothelium) were grouped and visualized using boxplots. To construct an energy score for each sample, the proteins in the oxidative phosphorylation complexes and citric acid cycle were standardized to have a mean energy score of 0 and variance of 1. Next, for each sample the standardized expression values were summed for all proteins except lactate dehydrogenase A and B and lactate. Their expression values were subtracted from the energy score, as these three proteins were expected to be upregulated in the mucosal samples of the map.

**Targeted metabolomic analysis**

After partitioning through ice-cold chloroform and water, organic and aqueous layers were carefully transferred into new glass vials. Proteins and lipids were removed from extracted samples using a 3K Amicon Ultra filter (Millipore, Burlington, MA, USA). Dried pellets were dissolved in methanol and water (50:50 v/v).

Chromatographic separation of extracted metabolites was performed using hydrophilic interaction chromatography and reverse-phase chromatography techniques. The metabolites were separated through an XBridge Amide high-performance liquid chromatography column (3.5 μm, 4.6 × 100 mm, Waters, Milford, MA, USA) in both electrospray ionization-positive and -negative mode. The details about the liquid chromatography methods were described in our previous publications [18,87,88]. The data were acquired via multiple reaction monitoring using a 6495 Triple Quadrupole mass spectrometer coupled with a high-performance liquid chromatography system (Agilent, Santa Clara, CA, USA) and Agilent MassHunter quantitative analysis software [18]. The acquired data were analyzed and integrated into each peak using MassHunter. The extracted peak area was log2 transformed and normalized to an isotopically labeled internal standard for each method.

In total, 92 metabolites were profiled and passed quality control. In addition to clustering and visualizing individual metabolites across samples using heatmaps, the relative metabolic pathway activity level in each sample was calculated using single-sample GSEA. Specifically, 74 metabolic pathways were extracted from the KEGG database. Single-sample GSEA is an extension of GSEA in that it compares the difference in empirical cumulative distribution functions of metabolites’ ranks inside and outside a given metabolic pathway to calculate an ES for each sample. Single-sample GSEA ESs could reflect how the metabolites in a pathway are coordinately upregulated or downregulated within a sample, with further normalization across all samples. How the pathways differed among the NU/LGIN, HGIN, and UC groups was examined using hierarchical clustering and heatmaps.

**Integrative analysis of pathways**

For integrative analysis of pathways, the enrichment *p*-values for Ingenuity Pathway Analysis canonical pathways were computed based on a one-sided Fisher’s exact test for each of the four gene groups identified on RNA-seq, methylation, and mutational platforms (α and β mutational clusters). Because changes in the methylation patterns appeared to be dominant in the field effects, the nominally significant pathways with methylation enrichment *p*-values less than 0.05 were identified. To perform integrated analysis of the pathways across the platforms, the enrichment *p*-values for gene expression and mutation data for the same pathways were cross-checked with the methylation enrichment *p*-values and visualized using bar plots of −log10(*p*-value). This provided an integrated list of 73 monotonically dysregulated pathways in the field effects.

**Statistical analyses**

The Wilcoxon rank sum test was used for two-sample comparisons, whereas the Kruskal–Wallis test was used for multiple group comparisons. A chi-square test was used to examine associations between categorical variables; the Fisher’s exact test was used when the cell count was less than five in a given contingency table. All statistical tests were two-sided unless otherwise specified. The FDR was used to correct for multiple hypothesis testing. An adjusted *p*-value with an FDR less than 0.05 was considered significant unless otherwise specified. An unadjusted *p*-value less than 0.05 was considered nominally significant.

**
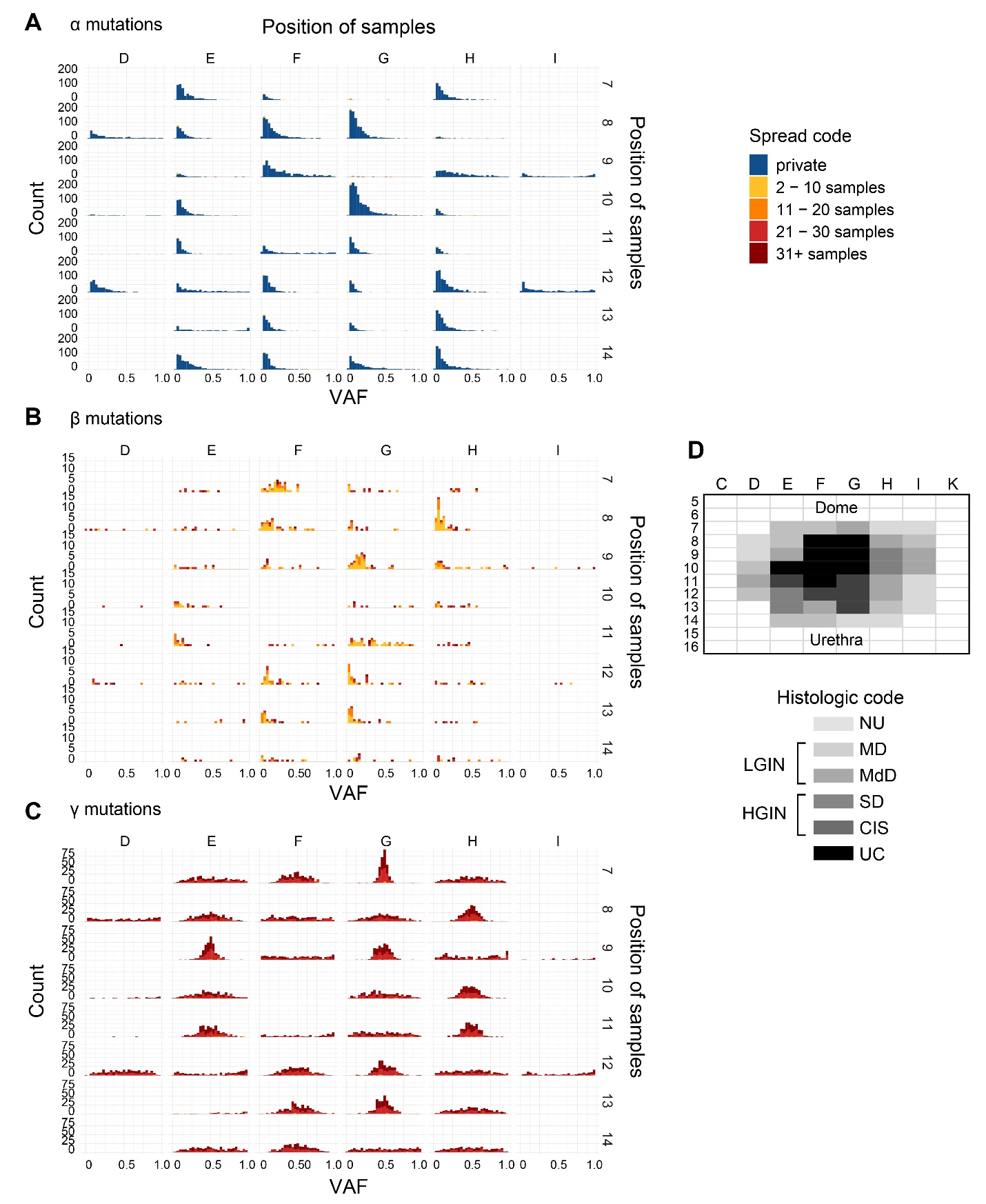
**

**Figure S1. Histograms of VAFs of α, β, and γ mutations in individual cystectomy samples.** (A) Histogram of α mutations, which are purely private and have right-skewed VAF distributions. (B) Histogram of regionally spread β mutations, with some having right-skewed VAF distributions and others being almost uniformly distributed. (C) Histogram of widespread γ mutations with mostly binomial-like unimodal distribution. (D) Histologic map of the cystectomy showing the positions of individual mucosal samples for A, B, and C.


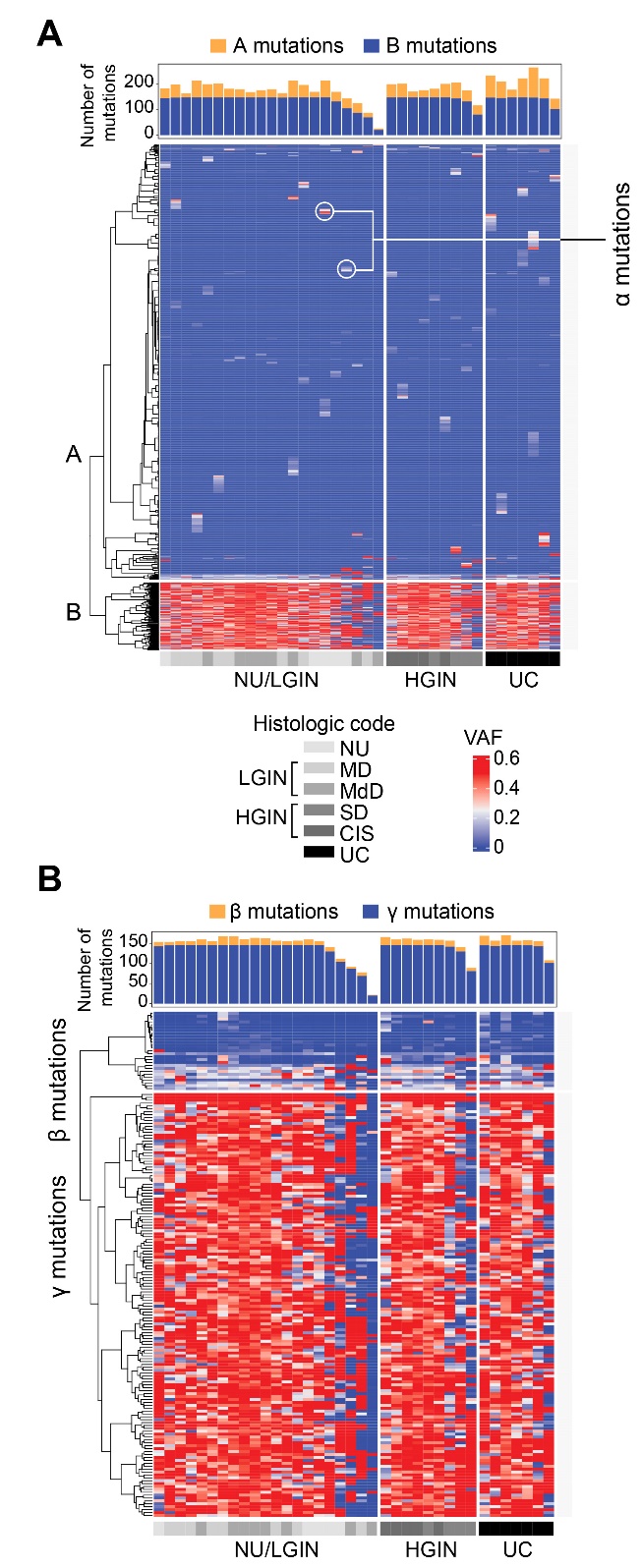


**Figure S2. Mutational landscape of bladder cancer evolution from field effects after filtration for COSMIC mutation.** (A) Heatmap of COSMIC mutations showing their VAFs in individual mucosal samples. The number of mutations in individual mucosal samples are shown in the top diagram. The heatmap of mutations showing their VAFs in individual mucosal samples before their filtration for COSMIC mutations in shown in Figure 2A. (B) Heatmap of COSMIC mutations ≥ 0.01 in genes showing variant alleles in at least three mucosal samples. The numbers of mutations in individual mucosal samples are shown in the top diagram. The heatmap of the same mutations before their filtration for COSMIC” mutations in shown in figure 2B.


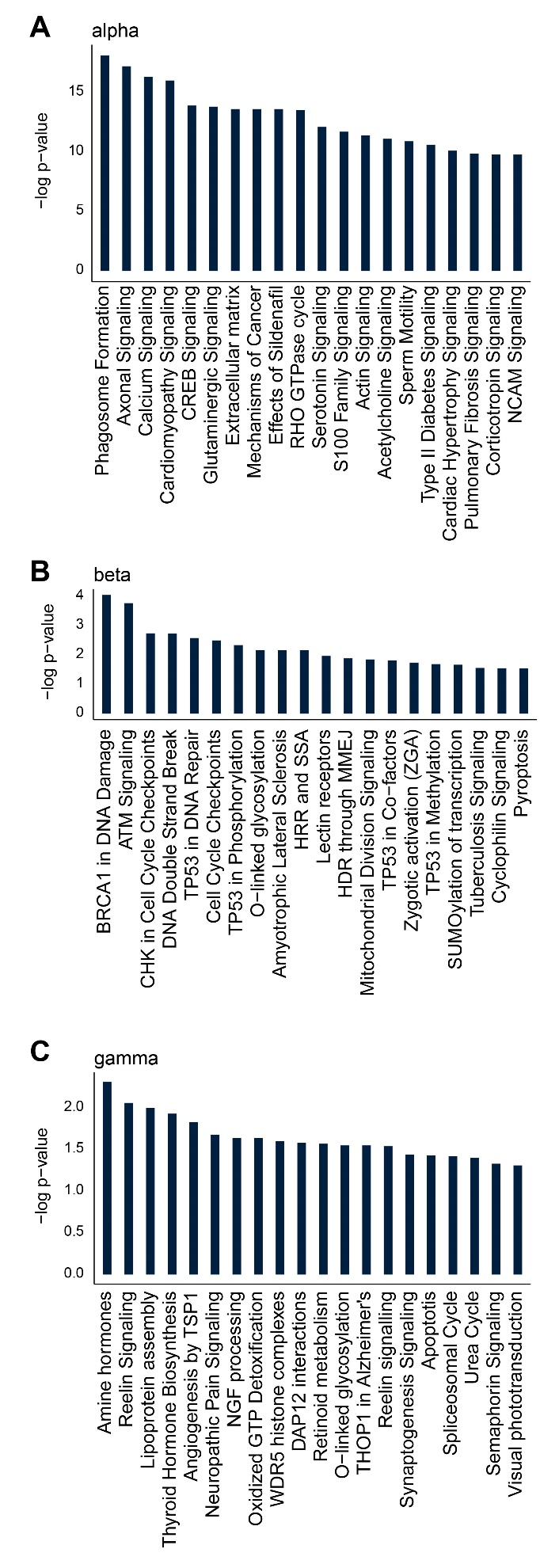


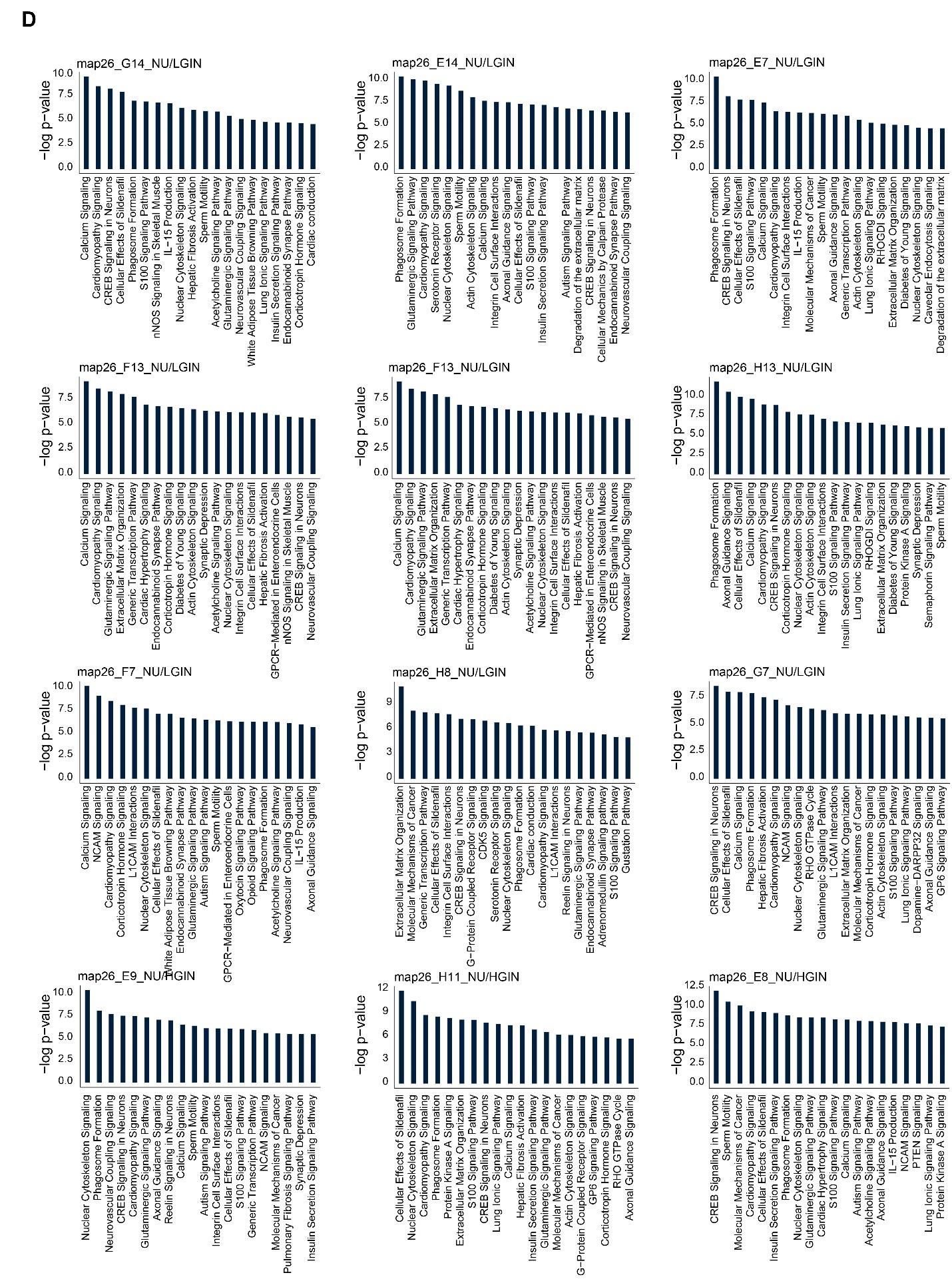


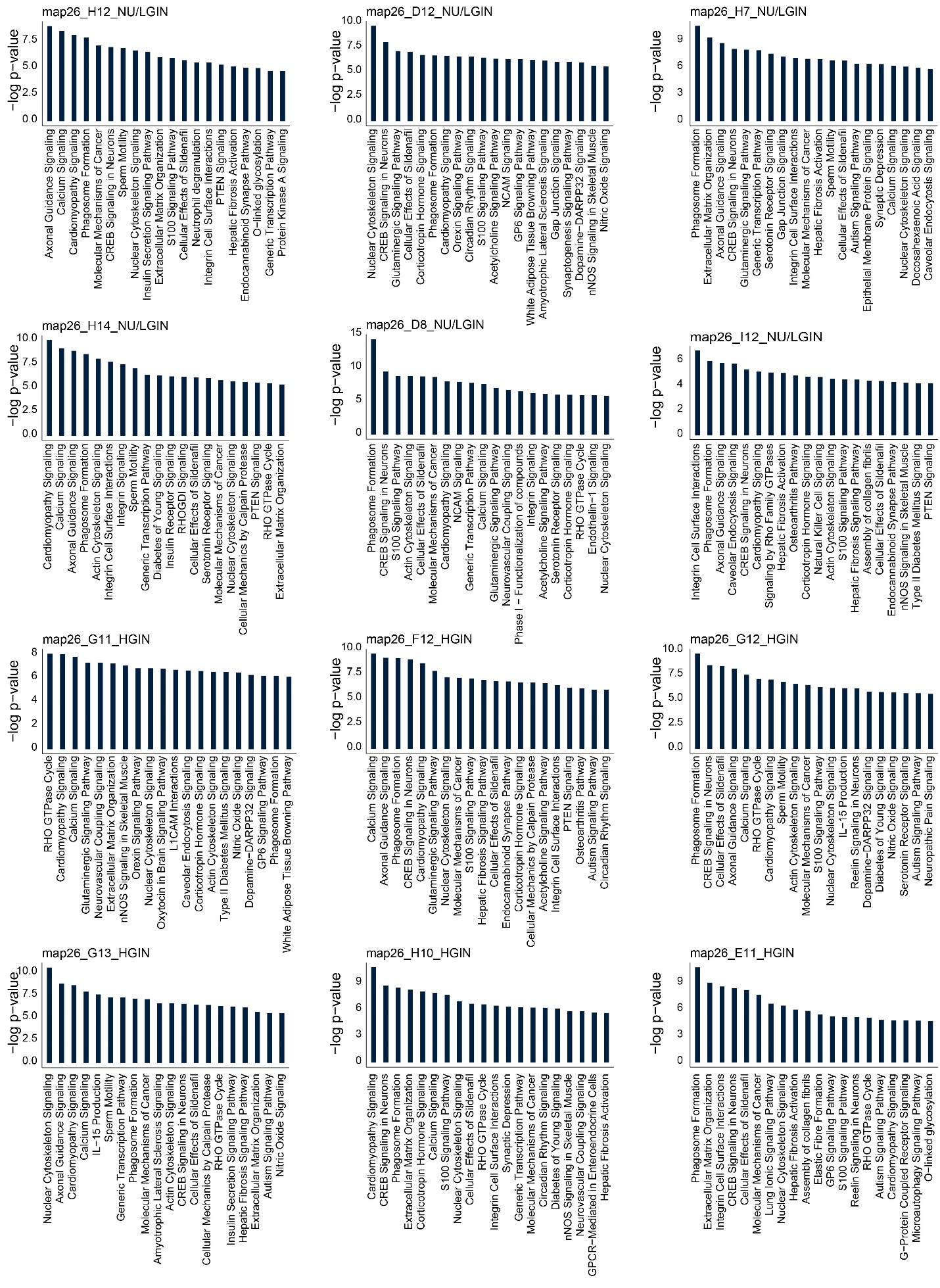


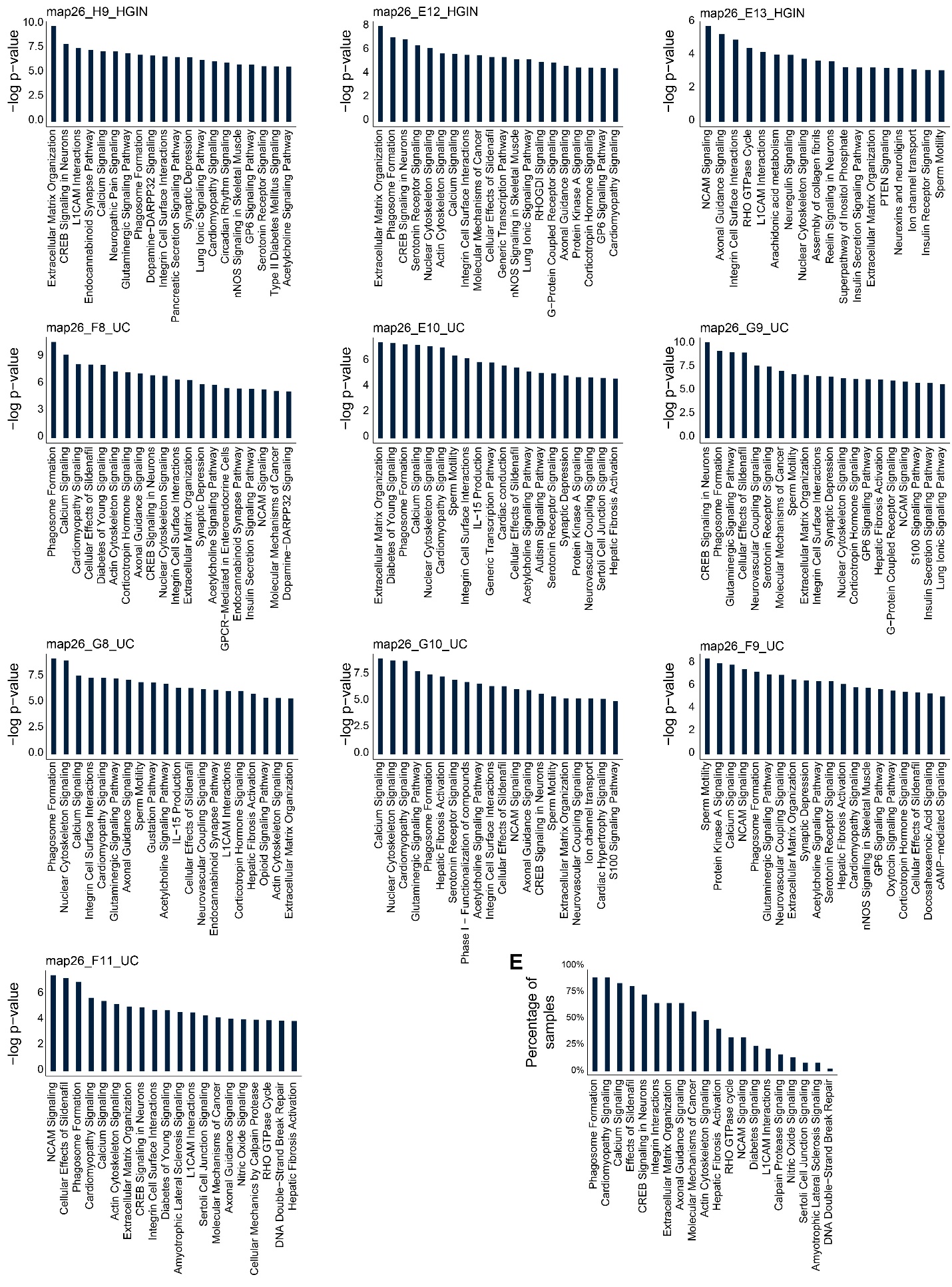


**Figure S3. Analysis of pathways affected by α, β, and γ mutations after filtration for COSMIC mutations.** (A) Top 20 dysregulated pathways affected by α mutations based on the combined analysis of all α mutations. (B) Top 20 dysregulated pathways affected by β mutations. (C) Top 20 dysregulated pathways affected by ϒ mutations. (D) Top 20 dysregulated pathways affected by α mutations in individual mucosal samples. (E) Frequency of involvement of top 20 dysregulated pathways in individual mucosal samples affected by α mutations.

**
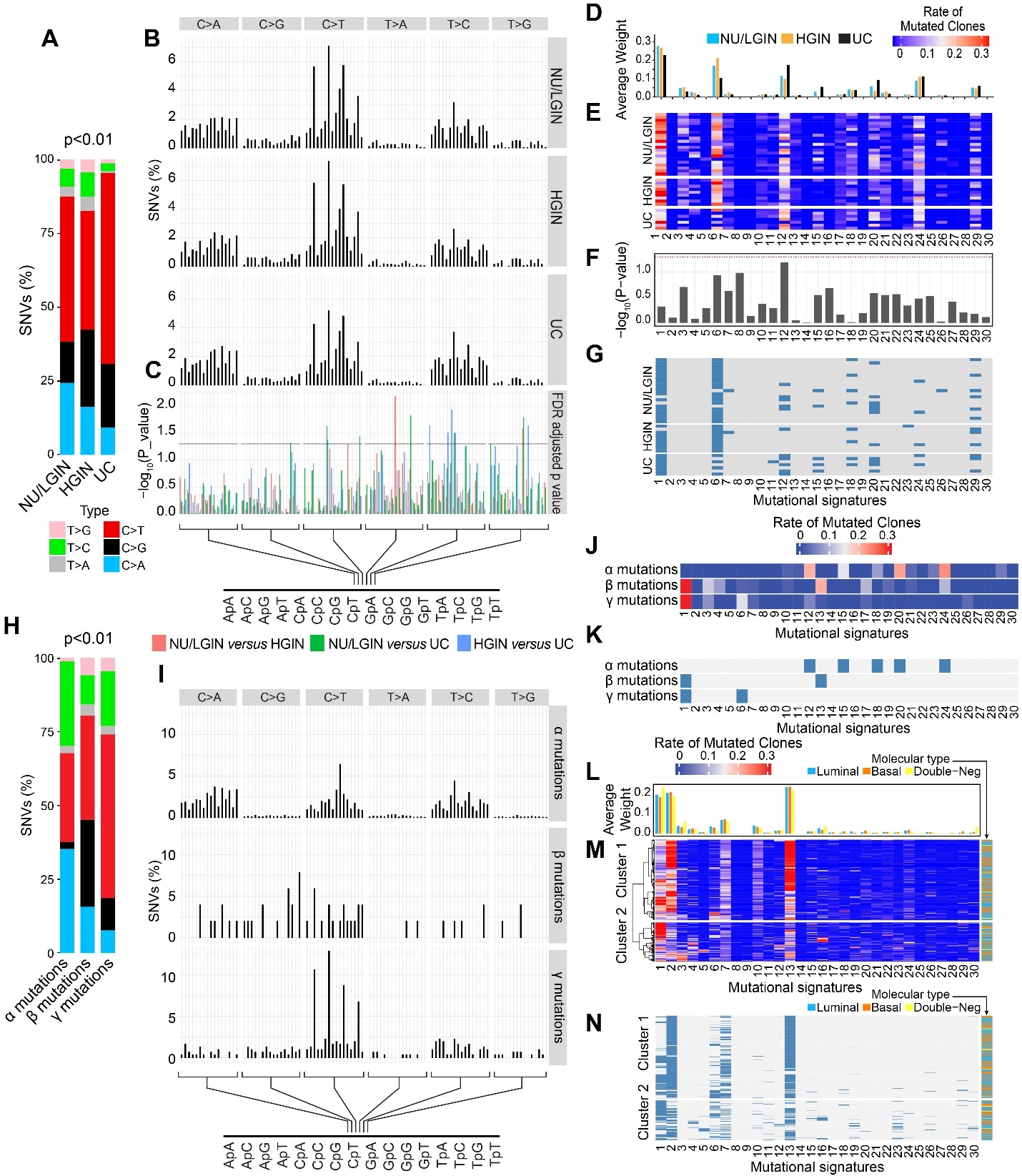
**

**Figure S4. Mutational signature of bladder cancer evolution from mucosal field effects.** (A) Bar graph of the distribution of all nucleotide substitutions in relation to cancer evolution from NU/LGIN through HGIN to UC. It shows an increase in the number of C > T mutations (*p* < 0.01 [Fisher’s exact test]) in the progression to HGIN and UC. (B) Proportions of SNVs in nucleotide motifs for each category of substitution in sets of mucosal samples corresponding to NU/LGIN, HGIN, and UC. (C) False discovery rates (FDRs) for nucleotide motifs in the progression of neoplasia from NU/LGIN through HGIN to UC. (D) Weight scores for mutagenesis signatures in mucosal samples corresponding to NU/LGIN, HGIN, and UC. (E) Weight scores for mutagenesis signatures in individual samples of bladder mucosa. (F) Significance of mutational signatures in the progression of neoplasia from NU/LGIN through HGIN to UC. (G) Significance of contributions to mutagenesis signatures in individual mucosal samples after bootstrapping. The blue boxes indicate *p*-values less than 0.05. (H) Bar graph of the distribution of nucleotide substitutions among α, β, and γ mutations. (I) Proportions of SNPs in nucleotide motifs for each category of substitution for α, β, and γ mutations. (J) Weight scores for mutagenesis signatures for α, β, and γ mutations. (K) Significance of contributions of mutagenesis signatures associated with α, β, and γ mutations after bootstrapping. The blue boxes indicate *p*-values less than 0.05. For A and H, *p*-values were calculated using a test of proportions. For C and F, *p*-values were calculated using the Wilcoxon test and Kruskal-Wallis test, respectively. (L) Average weight scores of mutagenesis patterns in the TCGA cohort (*n* = 408). (M) Weight scores of mutagenesis patterns in individual tumor samples of the TCGA cohort. (N) Significance of contributions of mutagenesis signatures in individual tumor samples after bootstrapping. The blue boxes indicate *p*-values less than 0.05.

**
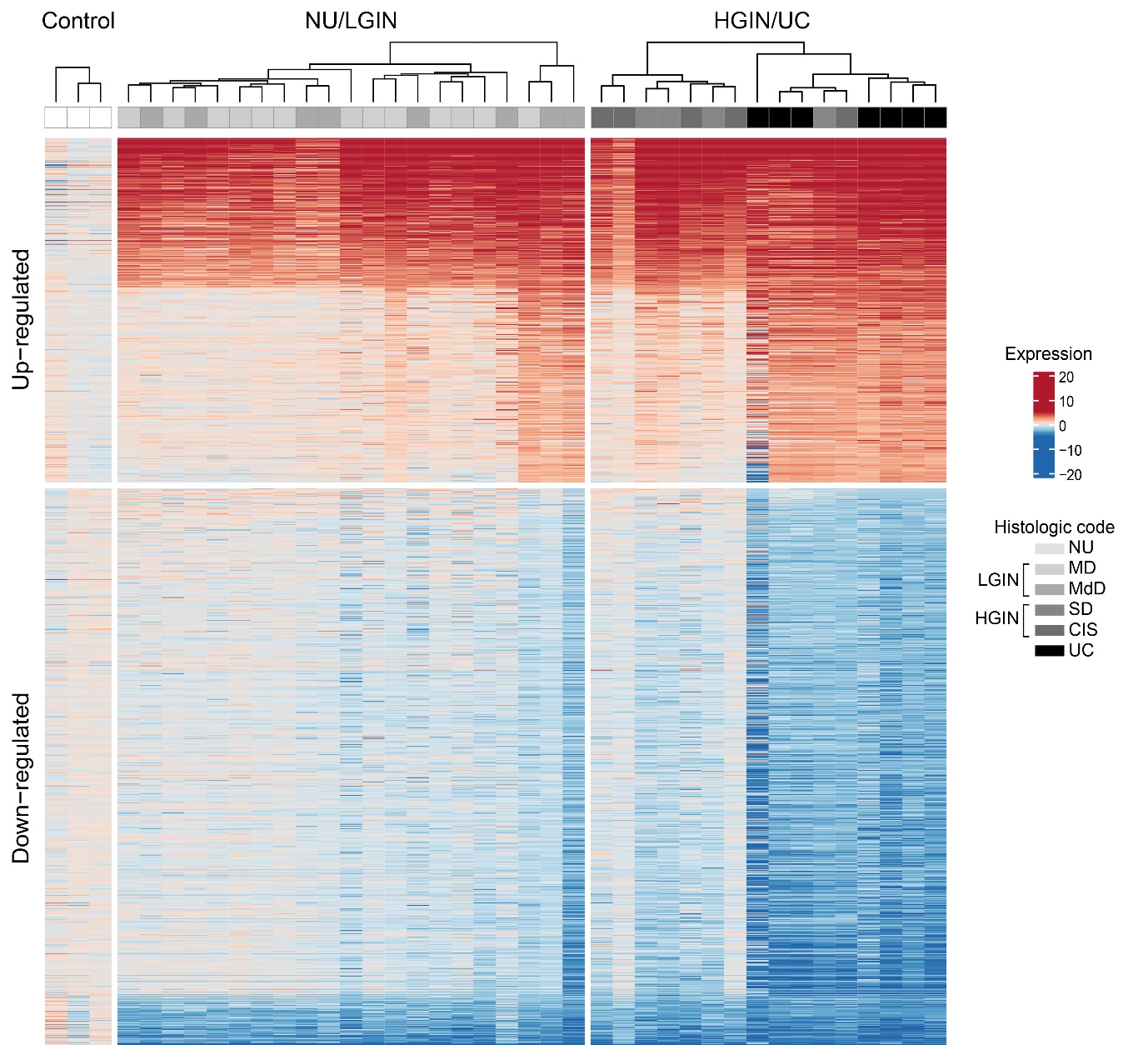
**

**Figure S5. Expression profile for monotonically dysregulated genes identified via RNA-seq of all mucosal samples from a cystectomy sample.**

**
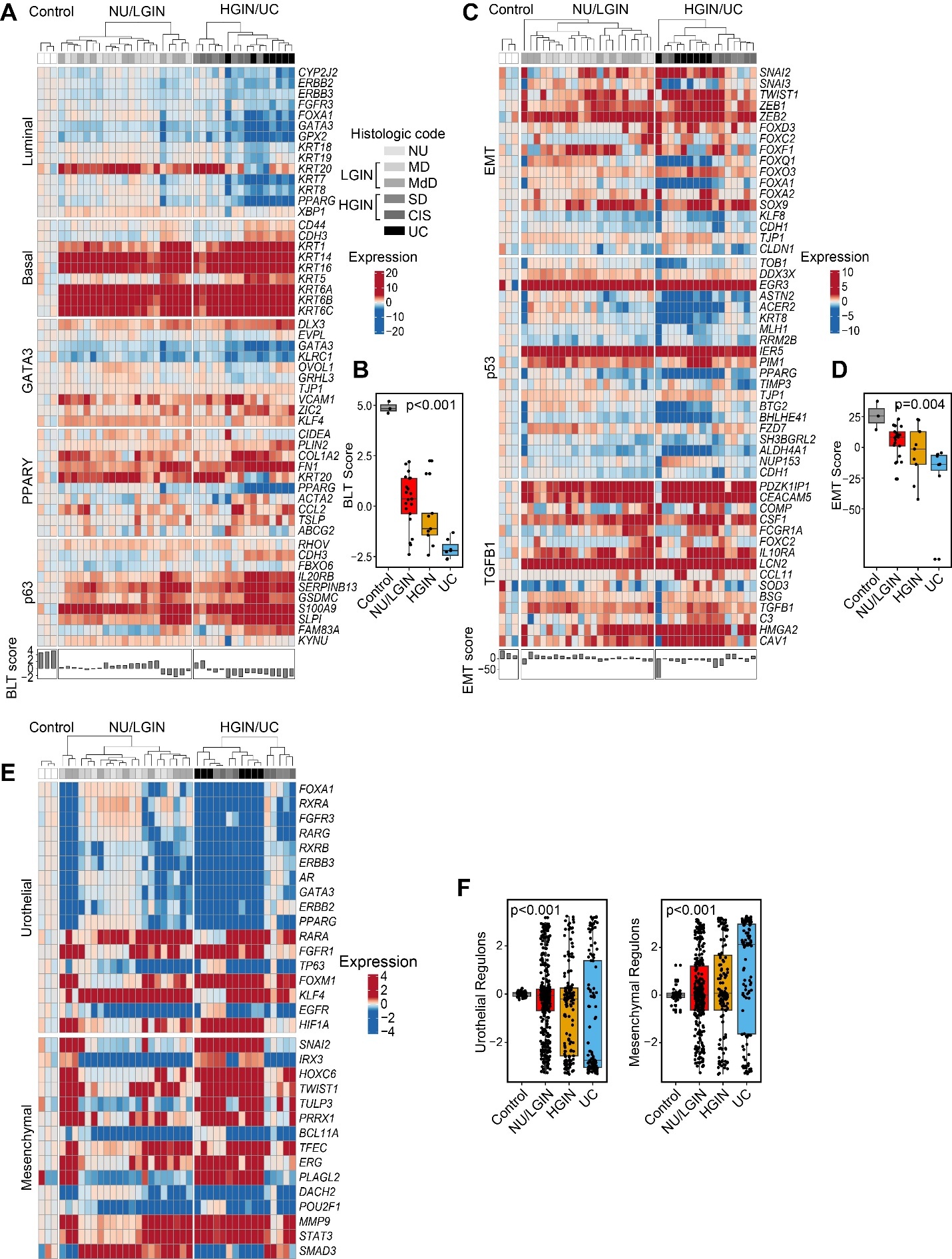
**

**Figure S6. Expression profiles for selected genes identified via RNA-seq of all mucosal samples from a cystectomy sample.** (A) Expression patterns for luminal and basal markers and BLT scores for all mucosal and tumor samples. (B) Boxplot of BLT scores for subsets of samples classified as NU/LGIN, HGIN, and UC. (C) Expression patterns for EMT markers and EMT scores for all mucosal and tumor samples. (D) Boxplot of EMT scores for subsets of samples classified as NU/LGIN, HGIN, and UC. (E) Activation scores for urothelial, neural, and mesenchymal neurons in all mucosal and tumor samples from the cystectomy sample. (F) Boxplot of activation scores for urothelial (left) and mesenchymal (right) neurons in subsets of samples classified as NU/LGIN, HGIN, and UC.

**
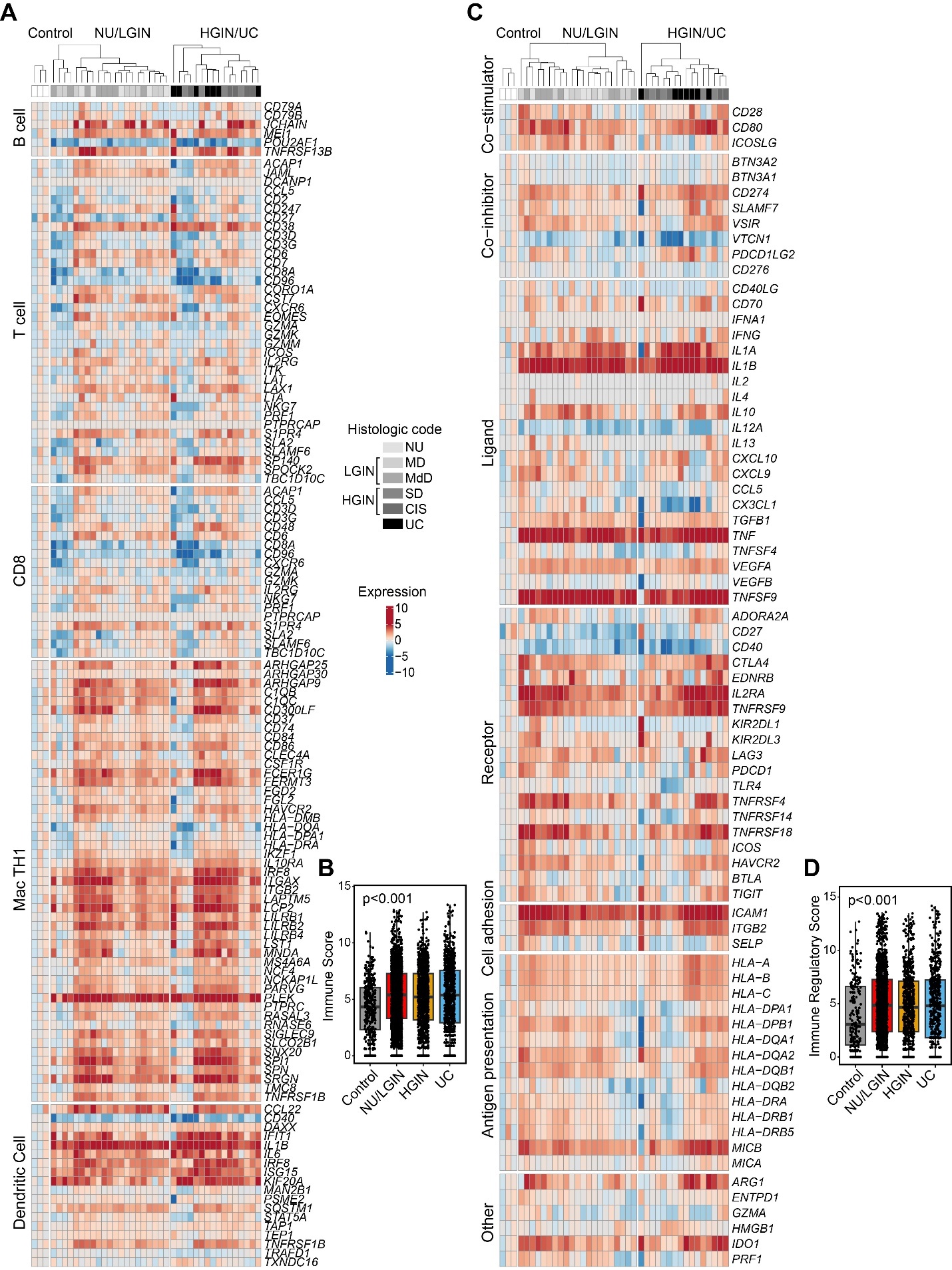
**

**Figure S7. Immune landscape of bladder cancer evolution from field effects.** (A) Expression profile for immune-related genes in all mucosal and tumor samples from a cystectomy sample. (B) Boxplot of expression scores for immune-related genes in subsets of samples classified as NU/LGIN, HGIN, and UC. (C) Expression profile for immunoregulatory genes in all mucosal and tumor samples from the cystectomy sample. (D) Boxplot of expression scores for immunoregulatory genes in subsets of samples classified as NU/LGIN, HGIN, and UC.


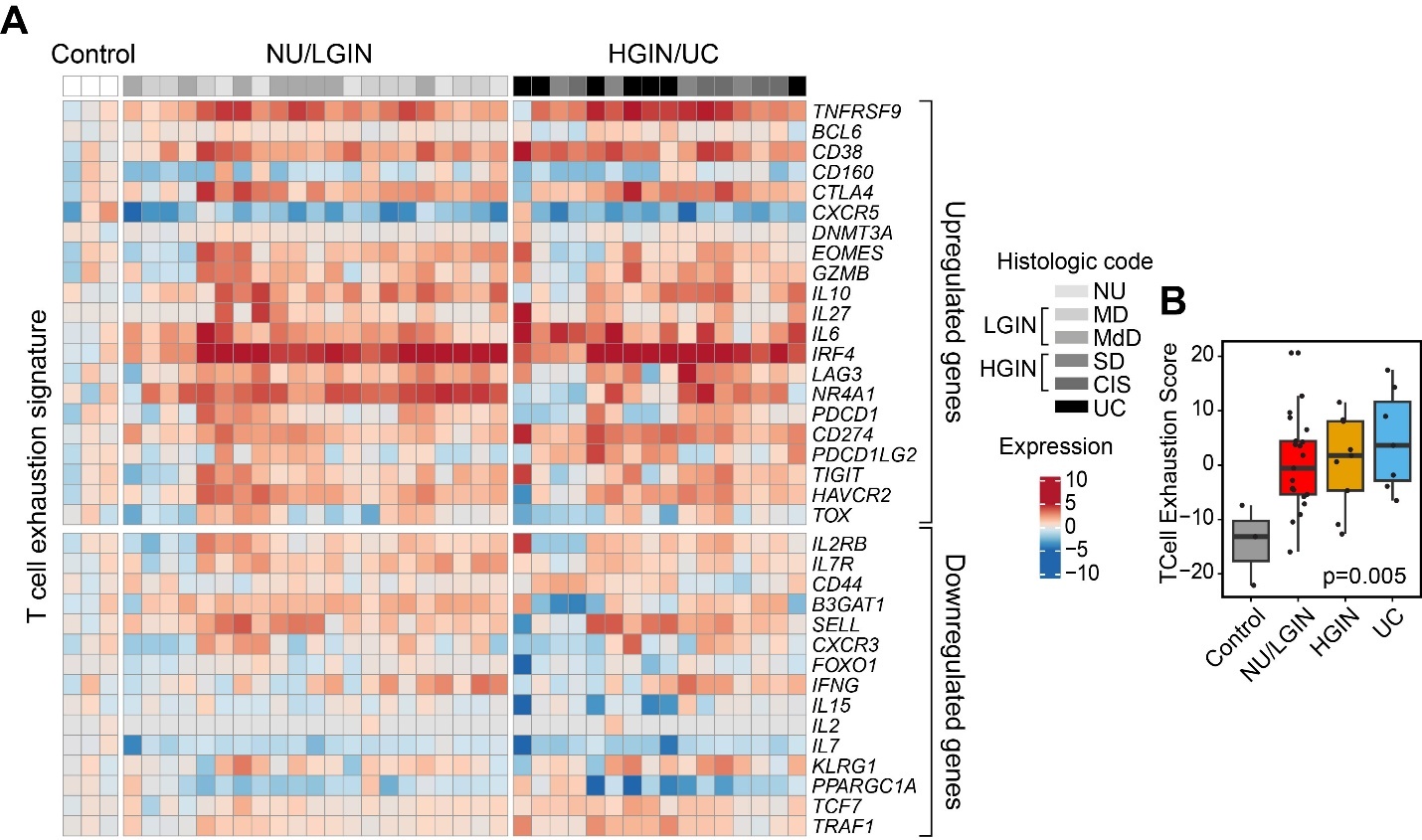


**Figure S8. T-cell exhaustion signatures in progression to bladder cancer from mucosal field effects.** (A) Expression profile of genes associated with T-cell exhaustion. (B) Boxplot analysis of T-cell exhaustion score for subset of mucosal samples classified as NU/LGIN, HGIN, and UC.


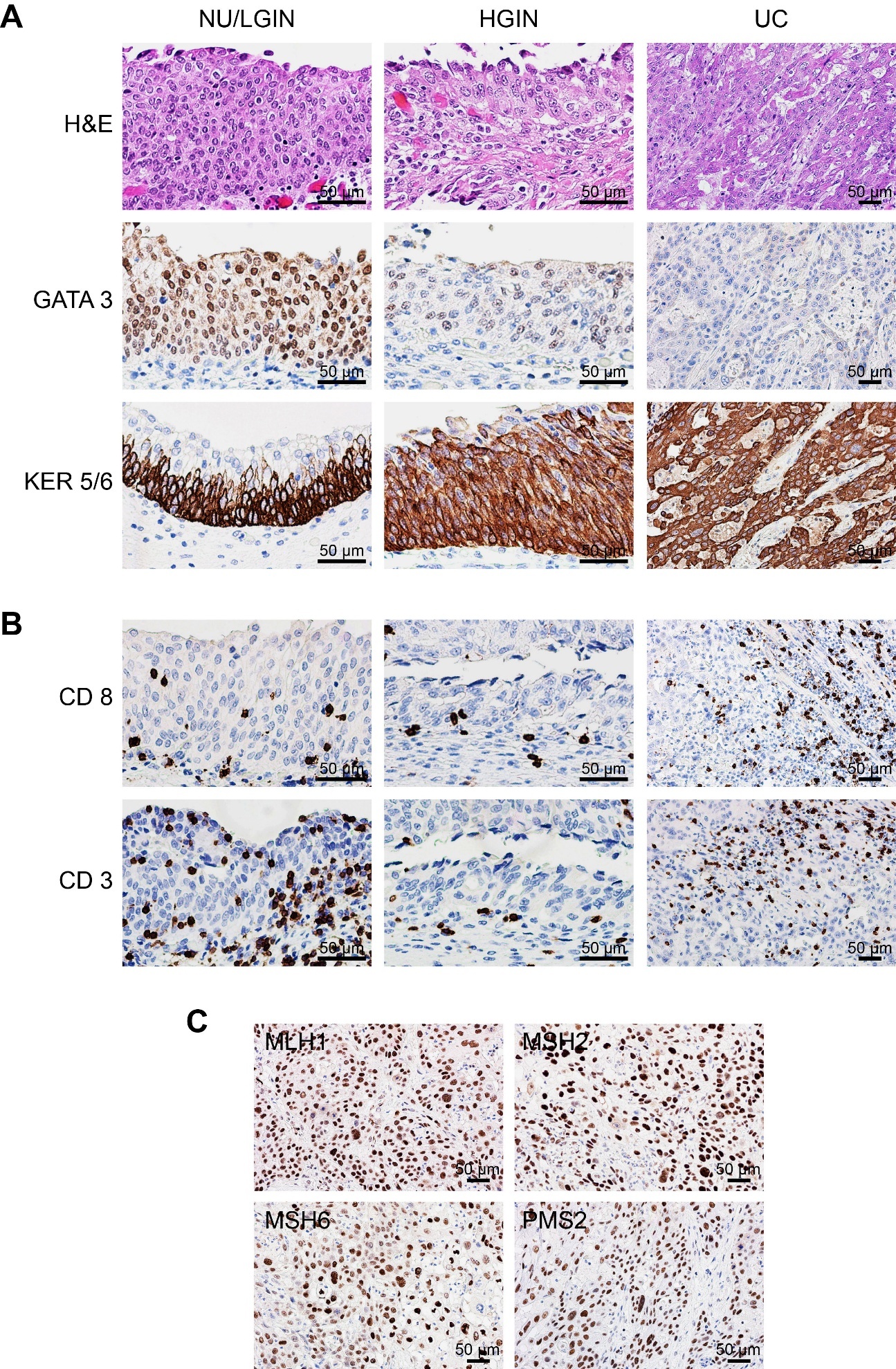


**Figure S9. Immunohistochemical validation of the basal field effects, immune infiltration, and retention of microsatellite stability gene.** (A) Immunohistochemical expression patterns of luminal (GATA3) and basal (KER5/6) markers in representative mucosal areas corresponding to NU/LGIN, HGIN, and UC. (B) Immune infiltrate of CD3 and CD8 positive T-lymphocytes in representative mucosal samples corresponding to NU/LGIN, HGIN, and UC. (C) Retentions of microsatellite stability proteins by immunohistochemical stains for MLH1, PMS2, MSH2, and MSH6.


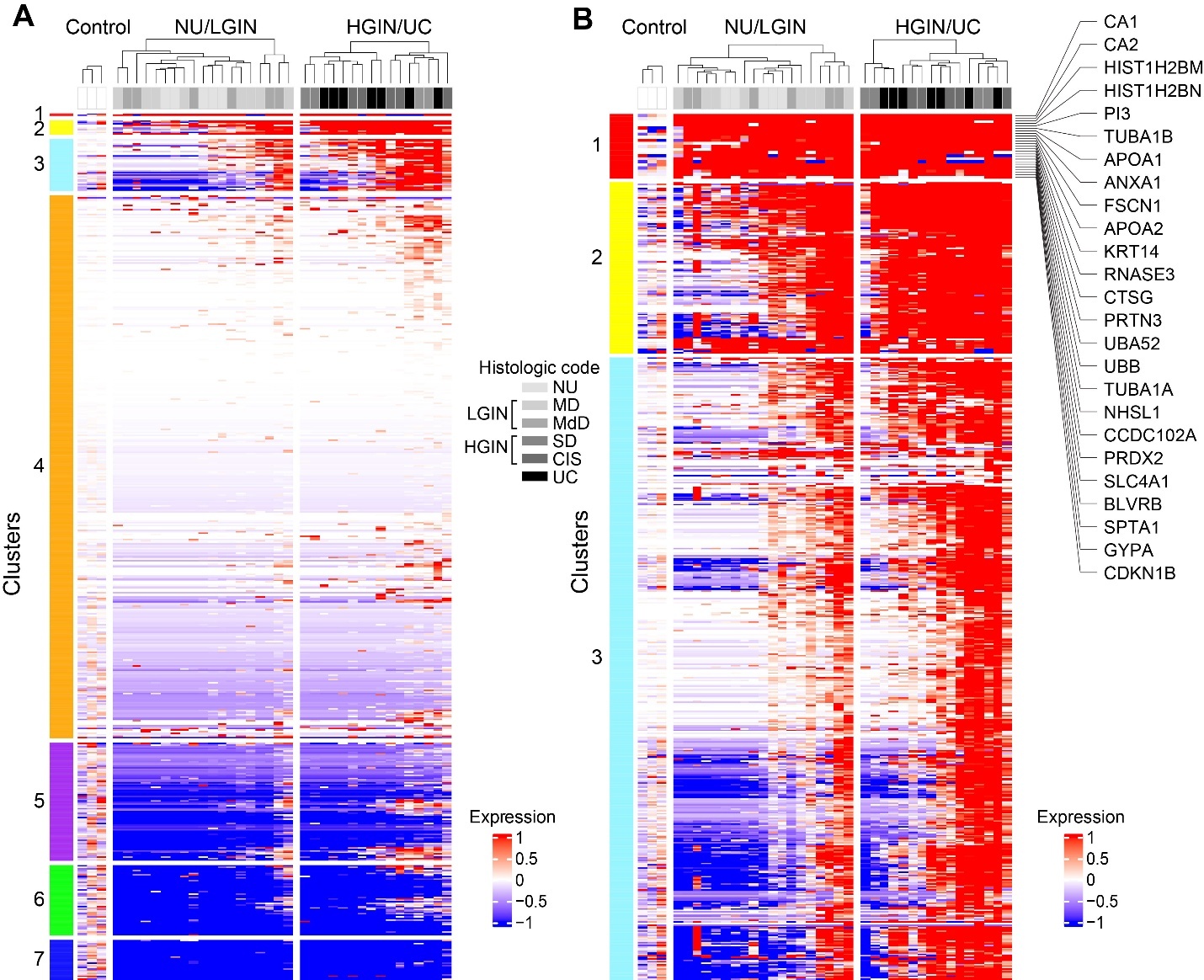


**Figure S10. Expression patterns for proteins identified by sequencing of all mucosal samples from a cystectomy sample.** (A) Heatmap of the expression patterns for all proteins in individual mucosal samples revealing seven distinct clusters. (B) Heatmap of enlarged clusters of overexpressed proteins (clusters 1–3 in A) upregulated in progression of bladder cancer from field effects.


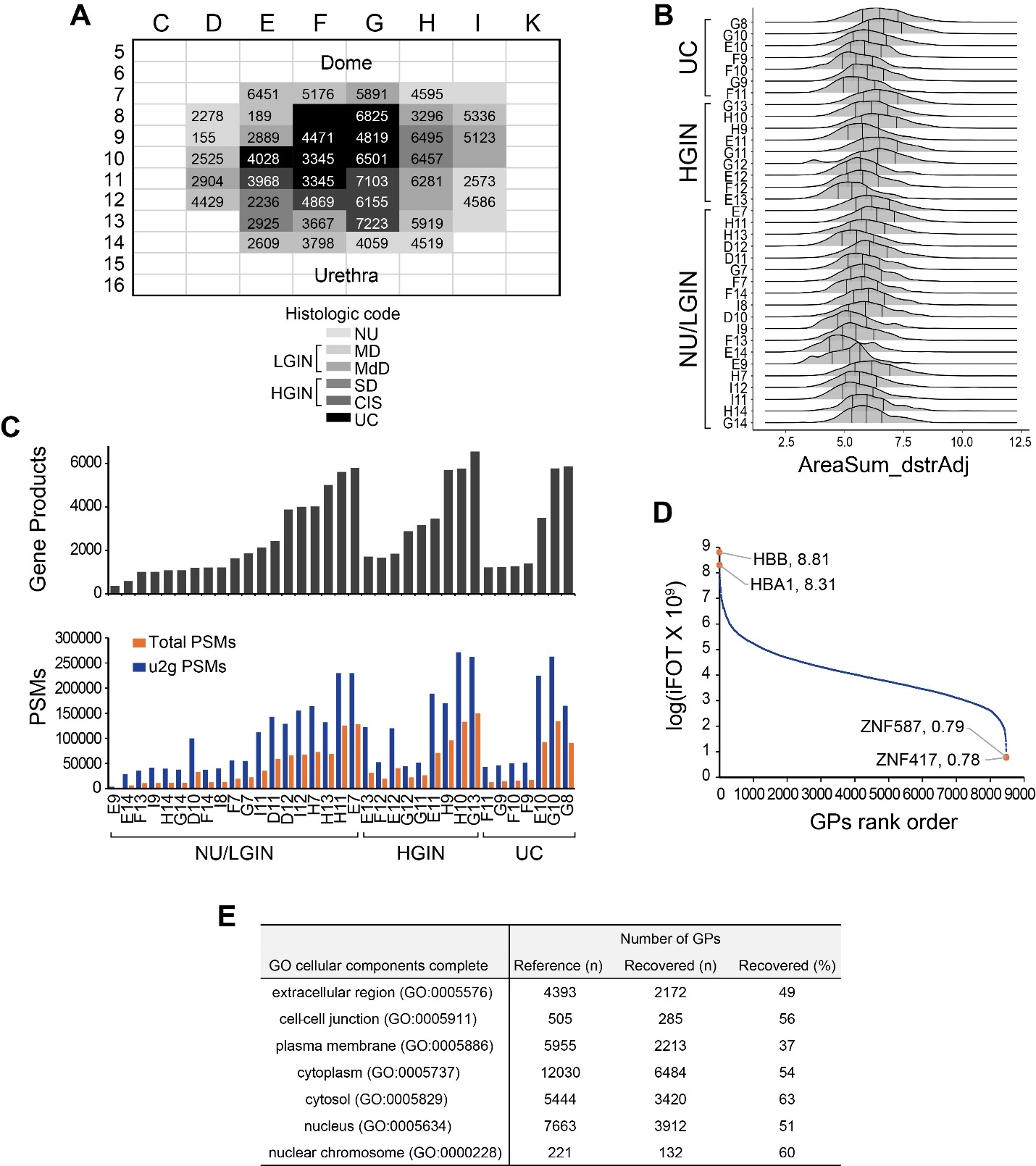


**Figure S11. Proteome profiling of a whole-organ map of a cystectomy sample obtained from a patient with bladder cancer.** (A) Whole-organ map of the cystectomy sample with superimposed numbers of proteins identified in individual mucosal samples. (B) Recovery of proteins from each individual mucosal sample from the cystectomy sample. (C) Identification of gene products and peptide spectrum matches in individual mucosal samples. (D) Distribution of the rank order of normalized protein abundances (iFOT) from the highest to the lowest. GPs, gene-protein products. (E) Numbers of GPs recovered from different cellular compartments. GO, Gene Ontology.


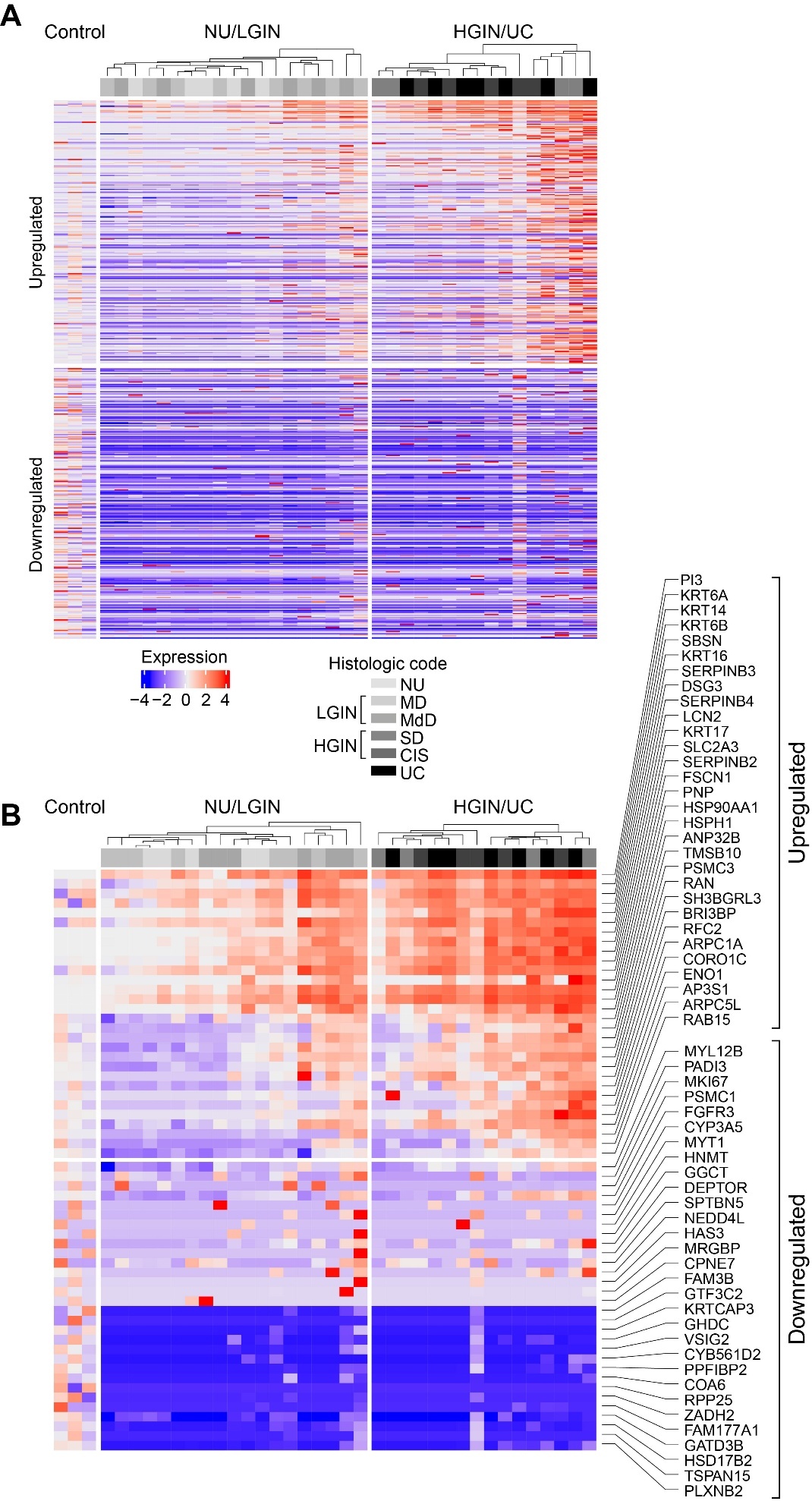


**Figure S12. Heatmap of proteins showing alterations in the same direction as their respective mRNA.** (A) Heatmap of expression pattern for 486 proteins showing upregulation or downregulation in the same direction as their respective mRNA in individual mucosal samples of the cystectomy. (B) Top 30 upregulated and downregulated protein from panel A showing synchronous dysregulation with their respective mRNA in individual mucosal samples of the cystectomy.


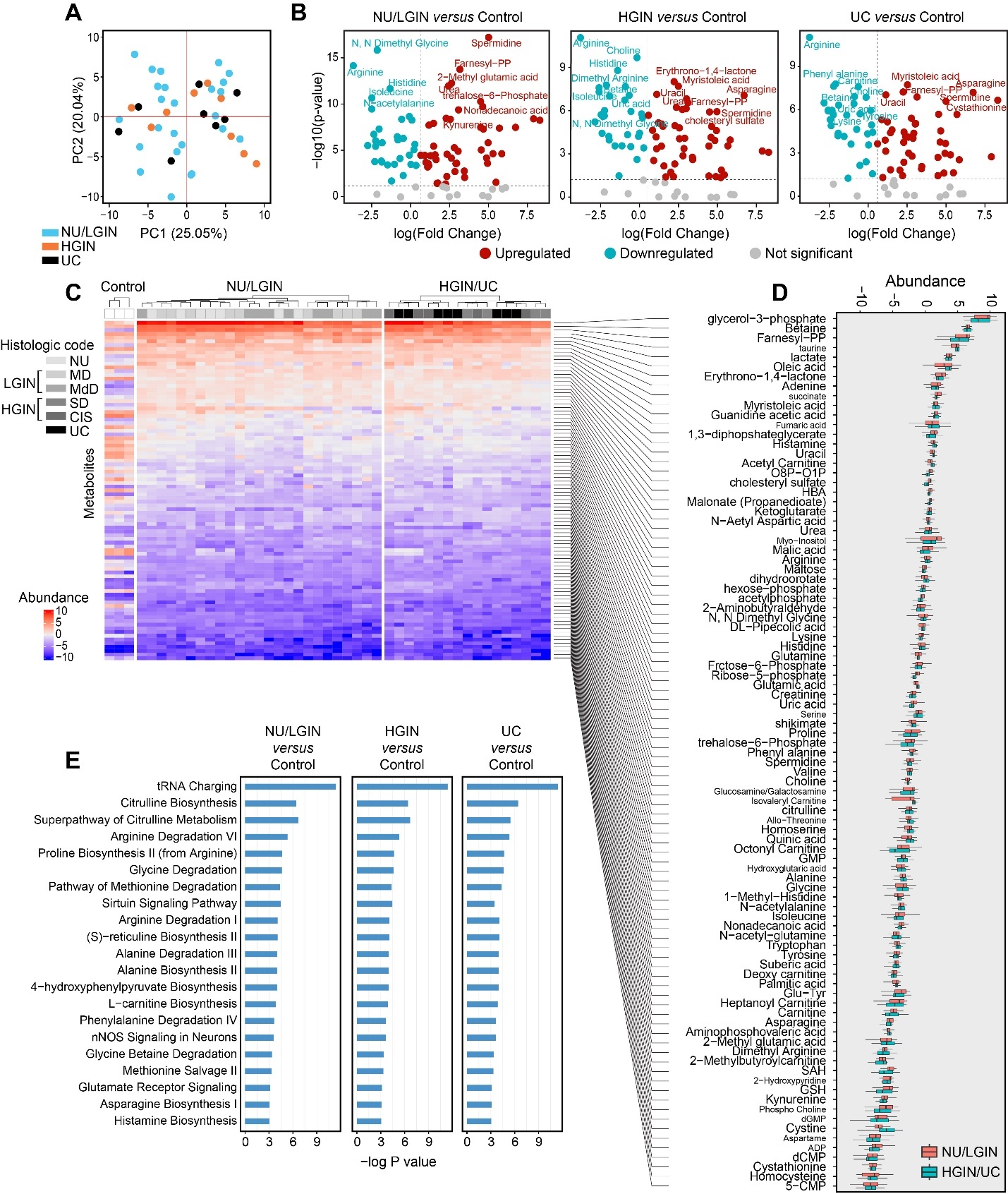


**Figure S13. Metabolomic profile of bladder cancer evolution from mucosal field effects.** (A) PCA of metabolite abundance data for all mucosal samples. (B) Volcano plots of all identified metabolites comparing log2 fold changes with −log *p*-values in NU/LGIN, HGIN, and UC samples *versus* those in control samples. (C) Heatmap of all identified metabolites in mucosal samples from a cystectomy sample. (D) Boxplot of the fold change in metabolite abundance in NU/LGIN and HGIN/UC samples compared with that in control samples. (E) Metabolomic KEGG pathways monotonically dysregulated in evolution of bladder cancer from field effects showing −log *p* values for the comparison of NU/LGIN, HGIN, and UC samples with control samples.


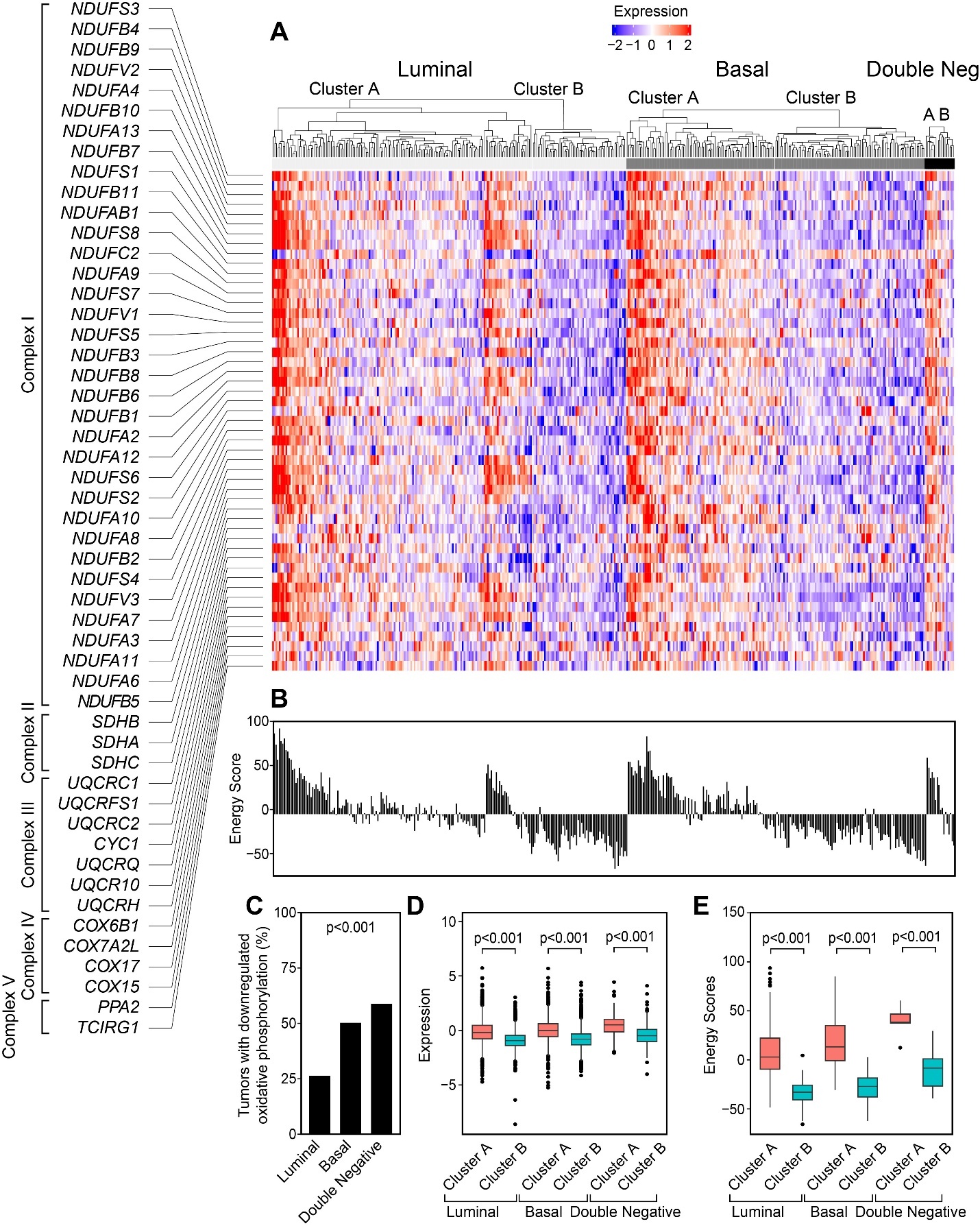


**Figure S14. Expression pattern of mRNA encoding enzymes involved in mitochondrial oxidative phosphorylation in the TCGA cohort (*n* = 408).** (A) Heatmap of expression levels of mRNA encoding enzymes of the mitochondrial oxidative phosphorylation complexes in individual samples of the TCGA cohort classified according to their molecular subtypes (luminal, basal, and double negative). (B) Energy scores in individual samples of the TCGA cohort corresponding to the heatmap shown in A. (C) Proportion of cases with the downregulation of mitochondrial oxidative phosphorylation in molecular subtypes of bladder cancer. (D) Boxplots of the expression levels of mRNA encoding enzymes of mitochondrial oxidative phosphorylation in clusters of molecular subtypes of bladder cancer. (E) Boxplots of energy scores in clusters of molecular subtypes of bladder cancer.


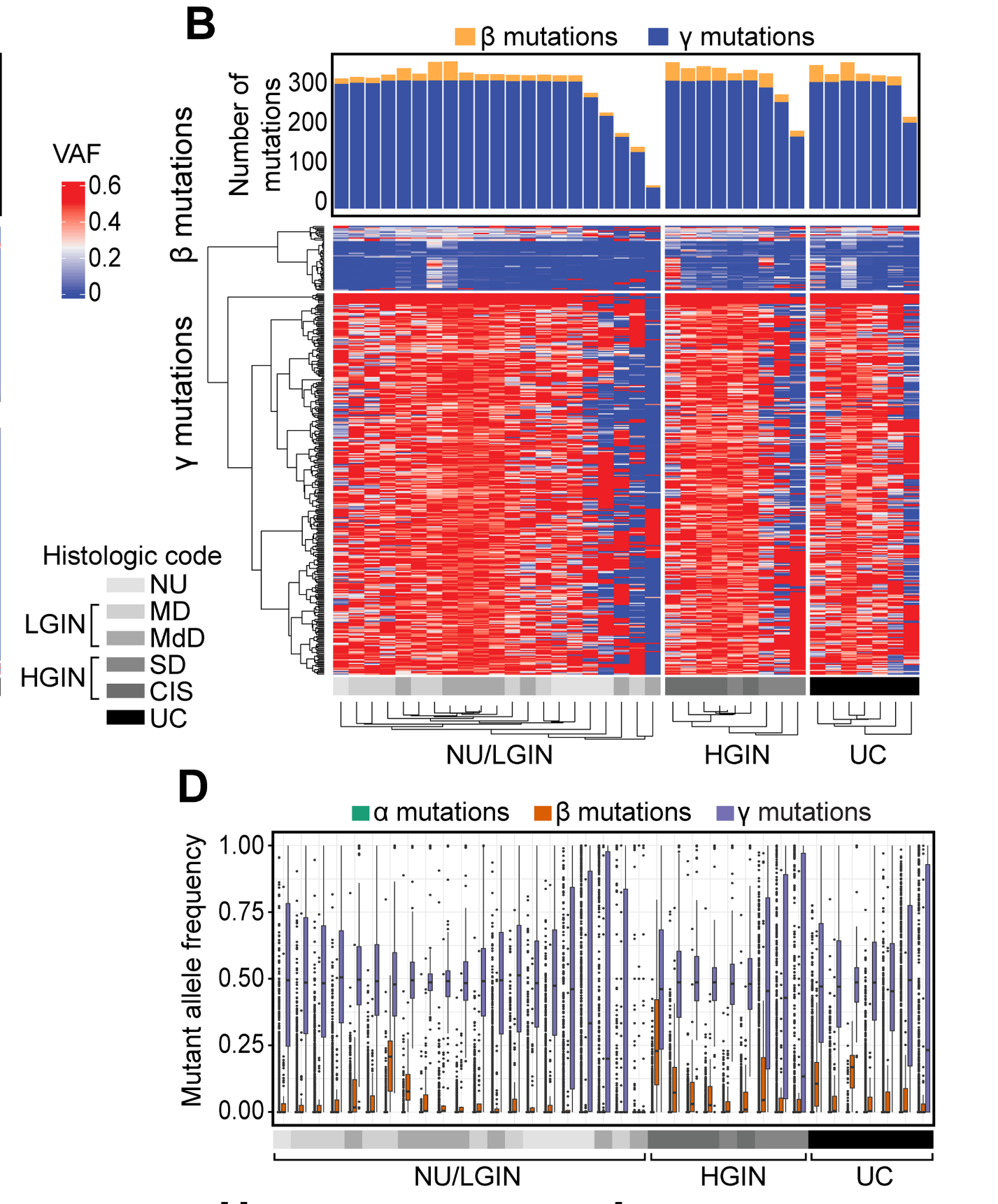


**Figure S15. Enlarged views of panels B and D from Figure 2.** (B) Heatmap of VAFs ≥ 0.01 in genes showing variant alleles in at least three mucosal samples. The numbers of β and γ mutations in individual mucosal samples are shown in the top diagram. (D) VAFs of α, β, and γ mutations in individual mucosal samples.


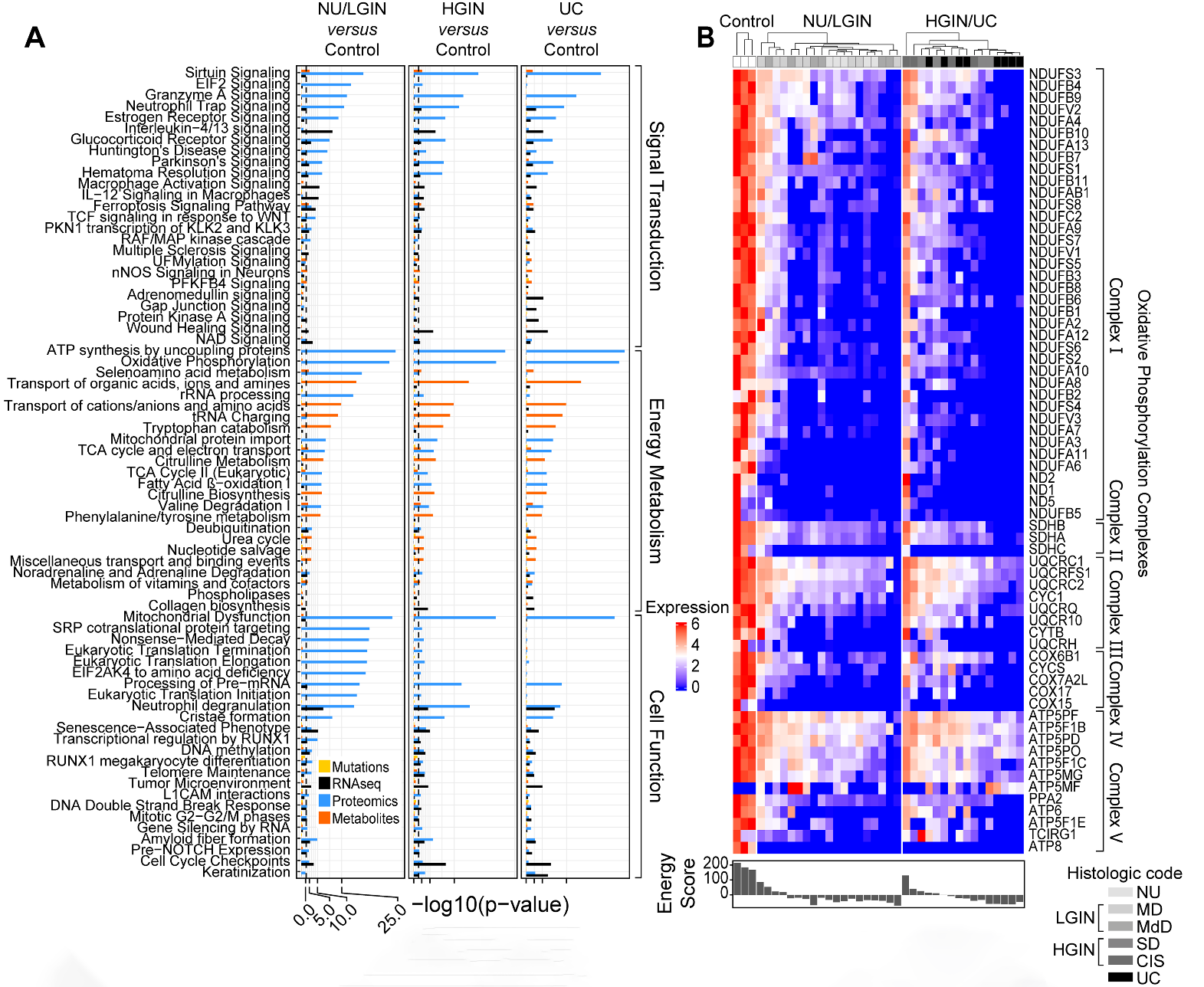


**Figure S16. Enlarged views of panels A and B from Figure 6.** (A) Combined analysis of monotonically dysregulated pathways in normal urethelium (NU)/low-grade intraepithelial neoplasia (LGIN), high-grade intraepithelial neoplasia (HGIN), and urothelial carcinoma (UC) (one-sided Fisher’s exact test *p*-value). (B) Proteomic expression levels for enzymes in mitochondrial oxidative phosphorylation complexes.

**Table S1.** Summary of mutations identified in the cystectomy specimen

|  | **Map 26** |
| --- | --- |
| **All mutations** |  |
| Nonsilent mutations | 12,764 |
| SNV | 12,022 |
| Insertions | 450 |
| Deletions | 292 |
|  |  |
| **Cluster α** |  |
| Nonsilent mutations | 12,431 |
| SNV | 11,698 |
| Insertions | 448 |
| Deletions | 285 |
|  |  |
| **Cluster B** |  |
| Nonsilent mutations | 333 |
| SNV | 324 |
| Insertions | 2 |
| Deletions | 7 |
|  |  |
| **Cluster β** |  |
| Nonsilent mutations | 54 |
| SNV | 51 |
| Insertions | 1 |
| Deletions | 2 |
|  |  |
| **Cluster γ** |  |
| Nonsilent mutations | 324 |
| SNV | 315 |
| Insertions | 2 |
| Deletions | 7 |

**Table S9.** Monotonically dysregulated proteomic pathways in progression of bladder cancer from field effects

| **KEGG Pathway Name** | **NU *versus* Control** |  | **HGIN *versus* Control** |  | **UC *versus* Control** |
| --- | --- | --- | --- | --- | --- |
| Thermogenesis | 1.00E−07 |  | 2.70E−07 |  | 3.00E−07 |
| Chemical carcinogenesis - reactive oxygen species | 1.10E−07 |  | 2.90E−07 |  | 5.80E−07 |
| Nonalcoholic fatty liver disease | 1.70E−07 |  | 6.60E−07 |  | 3.50E−06 |
| Retrograde endocannabinoid signaling | 1.50E−06 |  | 3.90E−06 |  | 6.50E−06 |
| Diabetic cardiomyopathy | 4.00E−06 |  | 4.30E−06 |  | 4.70E−06 |
| Metabolic pathways | 7.80E−06 |  | 7.80E−06 |  | 7.80E−06 |
| Valine, leucine and isoleucine degradation | 7.80E−06 |  | 7.80E−06 |  | 7.80E−06 |
| Fatty acid metabolism | 7.80E−06 |  | 7.80E−06 |  | 7.80E−06 |
| Ribosome biogenesis in eukaryotes | 7.80E−06 |  | 7.80E−06 |  | 7.80E−06 |
| Spliceosome | 7.80E−06 |  | 7.80E−06 |  | 7.80E−06 |
| Propanoate metabolism | 7.80E−06 |  | 7.80E−06 |  | 7.80E−06 |
| Fatty acid degradation | 7.80E−06 |  | 7.80E−06 |  | 7.80E−06 |
| Oxidative phosphorylation | 7.80E−06 |  | 7.80E−06 |  | 7.80E−06 |
| SNARE interactions in vesicular transport | 1.20E−05 |  | 1.30E−05 |  | 2.40E−05 |
| Beta-Alanine metabolism | 3.80E−05 |  | 5.50E−05 |  | 8.90E−05 |
| Carbon metabolism | 2.40E−03 |  | 9.80E−05 |  | 7.80E−06 |
| Parkinson disease | 1.30E−04 |  | 2.30E−04 |  | 1.00E−04 |
| Tryptophan metabolism | 1.60E−03 |  | 5.60E−04 |  | 8.90E−04 |
| Fatty acid biosynthesis | 5.40E−04 |  | 6.90E−04 |  | 9.40E−04 |
| Nucleocytoplasmic transport | 7.80E−04 |  | 7.20E−04 |  | 8.20E−04 |
| Amyotrophic lateral sclerosis | 1.10E−03 |  | 8.20E−04 |  | 4.70E−04 |
| Arginine and proline metabolism | 5.50E−03 |  | 2.60E−03 |  | 3.90E−03 |
| Huntington disease | 3.90E−03 |  | 2.80E−03 |  | 2.20E−03 |
| Lysosome | 6.70E−03 |  | 3.10E−03 |  | 6.20E−03 |
| Glycerolipid metabolism | 1.70E−02 |  | 3.40E−03 |  | 5.20E−03 |
| PPAR signaling pathway | 2.70E−03 |  | 3.50E−03 |  | 2.00E−03 |
| Histidine metabolism | 3.90E−03 |  | 4.80E−03 |  | 6.30E−03 |
| Citrate cycle (TCA cycle) | 4.40E−03 |  | 5.90E−03 |  | 2.80E−03 |
| Butanoate metabolism | 4.70E−03 |  | 6.10E−03 |  | 8.40E−03 |
| Pyruvate metabolism | 1.40E−02 |  | 7.50E−03 |  | 1.50E−03 |
| Glyoxylate and dicarboxylate metabolism | 3.50E−02 |  | 1.60E−02 |  | 2.20E−02 |
| Lysine degradation | 3.80E−02 |  | 2.20E−02 |  | 3.10E−02 |
| Glycosphingolipid biosynthesis | 2.50E−02 |  | 2.80E−02 |  | 3.20E−02 |
| Glycerophospholipid metabolism | 7.10E−02 |  | 2.90E−02 |  | 2.30E−02 |
| Fatty acid elongation | 2.90E−02 |  | 3.60E−02 |  | 4.60E−02 |
| Glycosylphosphatidylinositol anchor biosynthesis | 3.40E−02 |  | 4.00E−02 |  | 4.90E−02 |
| Peroxisome | 6.00E−02 |  | 4.60E−02 |  | 3.90E−02 |
| Alzheimer disease | 1.40E−02 |  | 4.90E−02 |  | 1.40E−02 |
| Biosynthesis of cofactors | 8.70E−02 |  | 5.90E−02 |  | 2.90E−02 |
| Pathways of neurodegeneration: multiple diseases | 6.00E−02 |  | 6.70E−02 |  | 3.60E−02 |
| Ferroptosis | 1.70E−01 |  | 1.70E−01 |  | 4.00E−02 |
| Glycolysis / Gluconeogenesis | 4.10E−01 |  | 1.70E−01 |  | 4.50E−02 |
